# Problematic Love Behaviors and Correlated Factors: A Systematic Review with Subgroup Meta-Analysis Including Gender/Sex Moderation

**DOI:** 10.1007/s10508-026-03420-6

**Published:** 2026-04-24

**Authors:** Magdalena Sánchez-Fernández, Nerea Almeda, Mercedes Borda-Mas

**Affiliations:** 1 Department of Psychology, Universidad de Cádiz, Puerto Real, Cadiz, Spain; 2 Department of Personality, Assessment and Psychological Treatment, Universidad de Sevilla, Seville, 41018 Spain

**Keywords:** Love addiction, Emotional dependence, Pathological love, Psychological factors, Relationship science

## Abstract

Problematic love behaviors (PLB) represent a growing concern in relationship research, yet the field lacks a systematic synthesis that clarifies their conceptual and correlational landscape. This study addressed the pronounced conceptual heterogeneity in the literature by reviewing and meta-analytically integrating findings on PLB correlates across distinct conceptual domains. A systematic literature review was conducted following PRISMA guidelines, sourcing studies from Web of Science, Scopus, PsycInfo, and Medline. A total of 102 studies met the inclusion criteria and were categorized into three conceptual domains: manic love (ML; *n* = 55), emotional dependence (ED; *n* = 34), and love addiction (LA; *n* = 13). Quality assessment and narrative synthesis were performed, followed by subgroup meta-analyses for correlates supported by at least three studies within each domain. A subsequent moderation analysis by percentage of female participants was conducted to explain residual heterogeneity. Correlates were grouped into three categories: individual psychological, psychosocial, and partner-related factors. The meta-analyses revealed distinct correlational profiles: five significant correlates for ML, eleven for ED, and two for LA. Low self-esteem emerged as a shared correlate of ML and ED, whereas behavioral addictions were shared by ED and LA. The associations between ML and jealousy, as well as between ML and relationship satisfaction, were more pronounced in female-predominant samples. Conversely, the link between LA and anxious attachment was stronger in samples with a higher proportion of men. The systematic differentiation provided by this review has practical implications for establishing more consistent assessment criteria for PLB and advancing the understanding of their predictive factors.

Systematic Review Registration: PROSPERO; identifier: CRD42024513446.

**Supplementary Information:**

The online version contains supplementary material available at 10.1007/s10508-026-03420-6.

## Introduction

Romantic relationships are a fundamental aspect of human social life and are associated with greater mental and physical health (Braithwaite et al., 2010). However, some individuals experience difficulties in establishing healthy romantic bonds, which can lead to problematic ways of relating and subsequently have a negative impact on their well-being (South, 2023). This is why research has focused on the problematic aspects of romantic relationships (Fisher, 2014).

Problematic romantic relationship behaviors have had various conceptualizations in the literature. In Lee's (1973) theory of love styles, taken up by Hendrick and Hendrick's (1986) theory of love attitudes, it was proposed that mania or manic love style/attitude was characterised by obsession, dependence, preoccupation with rejection and/or loss of the partner, and exclusive dedication to one's partner (Karandashev, 2019). It has been suggested that this style is the most closely related to pathological love (Sophia et al., 2009; Stravogiannis et al., 2018).

On the other hand, other researchers refer to emotional dependency on partners with characteristics involving excessively intense, permanent, and dysfunctional emotional bonding, inability to end the relationship despite awareness of the negative balance, and prioritization of the other person over any activity or value, which generates interference in everyday life (Amor et al., 2022). The authors propose that this is not a case of dependent personality disorder, as individuals who are emotionally dependent on their partner may still demonstrate independence in other areas of their lives, such as socially or professionally. Instead, they suggested that emotional dependence on a partner is similar to an addictive disorder, where the relationship becomes a compulsive and central necessity in an individual's life (Camarillo et al., 2020).

Indeed, others have used the concept of love addiction to describe problematic behaviors in romantic relationships. They argue that these problematic ways of relating to a partner share characteristics with behavioral addictions (Earp et al., 2017; Fisher, 2014; Redcay & Simonetti, 2018; Reynaud et al., 2010; Sanches & John, 2019; Serebrisky, 2021; Sussman, 2010). Thus, love addiction is defined as a maladaptive or problematic pattern in love relationships, leading to clinically significant impairment or emotional distress (Earp et al., 2017; Reynaud et al., 2010; Sussman, 2010). This construct is characterized by clinical features similar to other addictions, such as the presence of a withdrawal syndrome in the absence of a loved one; a considerable amount of time spent in the relationship, either in reality or in thought; reduction of important social, professional, or leisure activities due to the relationship; persistent desire or unsuccessful efforts to reduce or control the relationship; continuation of the relationship despite the existence of problems caused by it; and the existence of attachment difficulties (Reynaud et al., 2010). Redcay and Simonetti (2018) proposed four assessment criteria for love addiction: impaired control, life impairment, disregard for the partner’s behavior, and prevention or reduction of undesirable or unbearable emotions. Costa et al. (2021), for their part, use the six elements of Grifiths' (2005) biopsychosocial model of addiction to conceptualise love addiction (i.e., salience, tolerance, mood modification, withdrawal, relapse, and conflict). It has been proposed that although it shares elements with other psychopathologies, it has its own conceptual framework that should be distinguished from other conditions (Sanches & John, 2019).

These three constructs (manic romantic attitudes, emotional dependence on partners, and love addiction) are collectively referred to in this study as Problematic Love Behaviors (PLB), given that they share common features such as obsessive and possessive feelings, difficulties in emotion regulation, and a high degree of partner dependence. Despite the use of the term “love” in these constructs, the study's focus is strictly on dyadic relationship dynamics and patterns of dependent or obsessive attachment toward a specific partner, thus differentiating them from anonymous or purely casual sexual encounters. While previous research has attempted to subsume all of these under a single overarching term (Cavalli et al., 2025; Özal et al., 2023; Sanches & John, 2019; Sussman, 2010), this approach has proven inadequate. The distinct theoretical origins and specialized assessment frameworks of ML, ED, and LA have instead led to conceptual confusion and fragmentation within the field, underscoring the need to treat them as separate, though related, phenomena.

Assessment tools also contribute to the conceptual fragmentation within PLB. Currently, there is a lack of a wide variety of scientifically validated instruments, which has made it difficult to establish consistent assessment criteria (Özal et al., 2023; Sussan et al., 2011). Therefore, a systematic review is essential to identify the instruments used for each specific problematic behavior and to assess which constructs are more consistently measured across the literature.

Previous research attempting to identify correlates of PLB has pointed to various personality traits–such as low self-esteem (Gori et al., 2023; Michalska et al., 2023; Olave et al., 2024), impulsivity (Etxaburu et al., 2024a; Olave et al., 2024), neuroticism (de Oliveira Santos & Diniz, 2024; Gori et al., 2024), psychopathological conditions–such as depression and anxiety (Campos-Arregui et al., 2023; Giacobbe et al., 2024) psychosocial factors–such as maladaptive familiar functioning (Etxaburu et al., 2024b), anxious attachment (Atlam et al., 2023; Gori et al., 2023), and partner-related variables–such as low relationship satisfaction (Soares et al., 2020) or dating-violence (Aiquipa-Tello et al., 2024; Granda Cabal & Moral-Jimenez, 2022; Jiménez-Cruz et al., 2022; Robles Ojeda et al., 2021)–as being associated with higher levels of PLB. However, these results are heterogeneous, and to date, no previous study has attempted to systematically synthesize these results while accounting for the specific underlying PBL, which is essential for advancing etiological clarity and developing tailored preventive strategies for each problematic behavior in romantic relationships.

It is essential to examine whether these correlational patterns differ according to gender/sex. Prior research has consistently documented gender/sex-related variations in the expression of problematic dynamics within romantic relationships. For example, research on attachment has shown that men typically present higher avoidance in close relationships, while women tend to display greater anxiety concerning intimacy and separation (Giudice, 2016). In the field of romantic jealousy, women and individuals with more feminine traits generally report experiencing more intense negative affective reactions, whereas men do not necessarily exhibit higher levels of aggressive responses (Banaszkiewicz, 2022). Studies on relational aggression indicate that women are more likely to acknowledge the use of indirect or relationally aggressive tactics, whereas men more often identify themselves as targets of such behaviors (Bitsola & Kyranides, 2021). Following conflict, men tend to emphasize aspects related to sexual availability, while women attach greater importance to emotional connection and reassurance. Evidence on maladaptive schemas further suggests gender/sex -specific pathways: for instance, women’s heightened fears of abandonment are linked to reduced relationship satisfaction for both partners, whereas men’s mistrust or abuse-related schemas appear to particularly undermine their partners’ satisfaction (Kover et al., 2024). In addition, gender/sex differences have been observed in the association between attachment and dating violence, with insecure attachment in men predicting more generalized violent behaviors, while in women it is more closely tied to sexual coercion (Choi & Park, 2025).

Taken together, these findings highlight the importance of investigating whether gender/sex moderates the associations between psychological correlates and problematic love behaviors (PLBs), as this could provide a more nuanced understanding of their underlying mechanisms.

### The Present Study

Given the critical lack of conceptual clarity and the high heterogeneity observed in the existing literature, the primary aim of the present study is to resolve this confusion through a differentiated analysis. The study conducts a systematic review and a subgroup meta-analysis to investigate the correlates of PLB separately across the three distinct domains: manic love, emotional dependence, and love addiction. The objectives of this study were as follows:

1. Examine the distinct conceptualizations and assessment instruments used for manic love, emotional dependence, and love addiction.
2. Review the specific correlates across the three domains.
3. Perform subgroup meta-analyses of established correlates.
4. Investigate the role of gender/sex as a potential moderating factor in the magnitude of the correlates' effect sizes.

## Method

### Literature Search

This study used four online databases, Web of Science, Scopus, PsycINFO, and Medline, and systematically searched for research literature related to PLB. The terms used were: (“love addiction” OR “pathological love” OR “addictive love” OR “relationship addiction” OR “affective dependenc*” OR “emotional dependenc*” OR “interpersonal dependenc*” OR “roman* addiction” OR “obsessive love” OR “maladaptive love” OR “relationship dependenc*” OR “love styles”). This search covered the research literature up to August 2025. The search was performed using the title and abstracts (Table 1).

**Table 1:** Search strategy and results by database

Database	Terms	Filters	N
Web of science	TI = ((“love addiction” OR “pathological love” OR “addictive love” OR “relationship addiction” OR “affective dependenc*” OR “emotional dependenc*” OR “interpersonal dependenc*” OR “roman* addiction” OR “obsessive love” OR “maladaptive love” OR “relationship dependec*” OR “love styles”)) OR AB = ((“love addiction” OR “pathological love” OR “addictive love” OR “relationship addiction” OR “affective dependenc*” OR “emotional dependenc*” OR “interpersonal dependenc*” OR “roman* addiction” OR “obsessive love” OR “maladaptive love” OR “relationship dependenc*” OR “love styles”))	Document types = article, Languages = English, French, Spanish, Italian	681
Scopus	TITLE-ABS (( "love addiction" OR "pathological love" OR "addictive love" OR "relationship addiction" OR "affective dependenc*" OR "emotional dependenc*" OR "interpersonal dependenc*" OR "roman* addiction" OR "obsessive love" OR "maladaptive love" OR "relationship dependenc*" OR "love styles"))	Document type = article, Languages = English, French, Spanish, Italian	874
Medline	title((“love addiction” OR “pathological love” OR “addictive love” OR “relationship addiction” OR “affective dependenc*” OR “emotional dependenc*” OR “interpersonal dependenc*” OR “roman* addiction” OR “obsessive love” OR “maladaptive love” OR “relationship dependec*” OR “love styles”)) OR abstract((“love addiction” OR “pathological love” OR “addictive love” OR “relationship addiction” OR “affective dependenc*” OR “emotional dependenc*” OR “interpersonal dependenc*” OR “roman* addiction” OR “obsessive love” OR “maladaptive love” OR “relationship dependenc*” OR “love styles”))	Document type = Journal article, Language = English, French, Spanish, Italian	322
PsycInfo	title((“love addiction” OR “pathological love” OR “addictive love” OR “relationship addiction” OR “affective dependenc*” OR “emotional dependenc*” OR “interpersonal dependenc*” OR “roman* addiction” OR “obsessive love” OR “maladaptive love” OR “relationship dependec*” OR “love styles”)) OR abstract((“love addiction” OR “pathological love” OR “addictive love” OR “relationship addiction” OR “affective dependenc*” OR “emotional dependenc*” OR “interpersonal dependenc*” OR “roman* addiction” OR “obsessive love” OR “maladaptive love” OR “relationship dependenc*” OR “love styles”))	Font type = scientific journal, Document type = journal article, Language = English, French, Spanish, Italian	664

### Inclusion and Exclusion Criteria

Studies that met the following criteria were included in this systematic review:

1. The study examined the correlation between PLB and other psychological outcomes (e.g., personality traits, attachment styles, or mental health variables).
2. The variables associated with PLB were assessed using a validated instrument that yielded a single continuous score.
3. The study involved community-based or non-clinical populations. This criterion was established to focus on the prevalence and correlates of PLB in the general population, avoiding the confounding effects of severe comorbid psychopathology and high treatment-seeking bias typical of clinical samples.
4. The study has been published in an academic journal.

Studies were excluded if:

1. They used a theoretical, qualitative, review, or single-case study design.
2. They did not provide sufficient statistical information to calculate or estimate a correlation coefficient.
3. No access to full article through institutional repositories.

### Selection of Articles

**Fig. 1:**
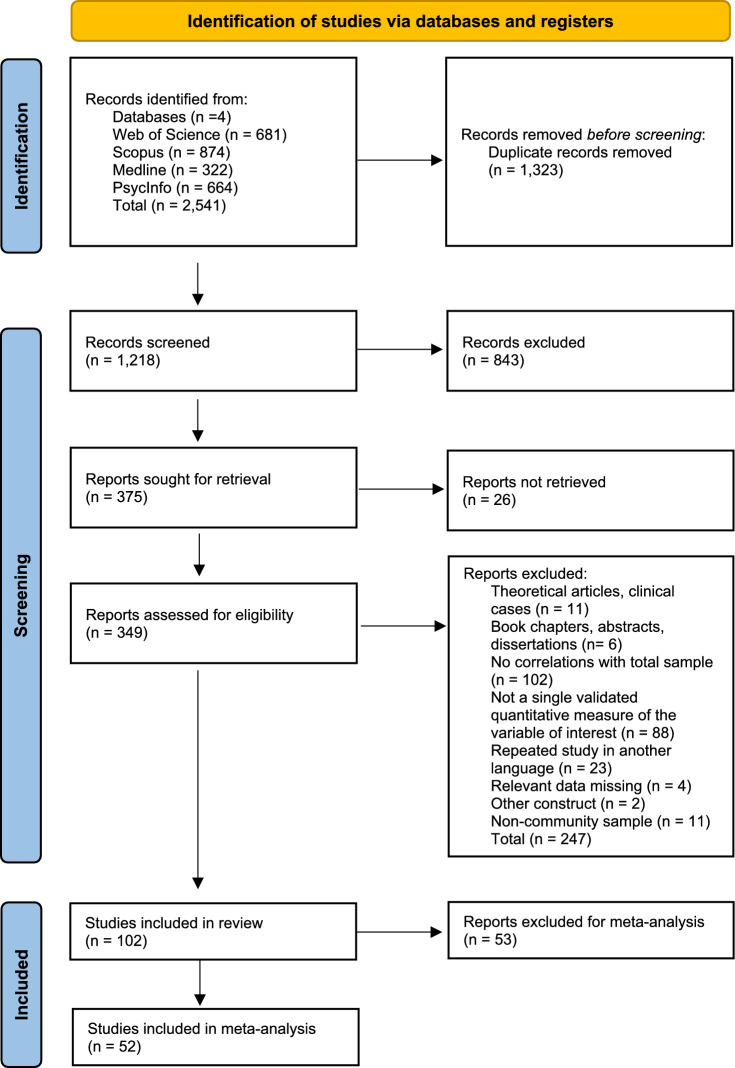
PRISMA diagram of study selection processes

This study adhered to the PRISMA protocol (Page et al., 2021) (Fig. 1). Of the 1,218 articles, 843 were excluded based on their abstracts or titles, and 349 were selected for a detailed review. The study selection process was conducted independently by two researchers.

Our initial sample consisted of 102 articles that met all the inclusion criteria and none of the exclusion criteria. The included studies were categorized into three main conceptual domains corresponding to specific PLBs: manic love (ML, *n* = 55), emotional dependence (ED,* n* = 34), and love addiction (LA, *n* = 13).

Subsequently, studies were selected for the meta-analysis based on the criterion that a specific correlate had to be supported by at least three independent studies. During this phase, if two articles reported data from the same study and measured the same correlation, only the article with the largest sample size was retained to ensure the independence of the effect sizes. As a result of this second screening, 52 articles were included in the final meta-analysis: 25 related to ML, 20 related to ED, and 7 related to LA.

### Data Extraction

From each of the 102 articles included in the systematic review, the following information was systematically extracted: sample characteristics (country, size, percentage of women, and age), specific conceptual domain studied (ML, ED, or LA), assessment instrument for the PLB, correlating factors analyzed, and direction of the relationship. For the 52 articles included in the meta-analysis, specific correlation coefficients (*r*) for each relevant correlate were extracted.

### Quality Assessment

Fourteen criteria of the "Quality Assessment Tool for Cohort and Cross-Sectional Observational Studies" (National Heart, Lung, and Blood Institute, 2014) were applied to assess each study. The second and third authors evaluated 52.4% of the articles (n = 43). After achieving inter-rater agreement, the second author conducted the quality assessment.

Four criteria (6, 7, 10, and 13) were not applicable to cross-sectional studies. Quality was assessed based on the judgement using the remaining 10 criteria (Table 2). The second criterion (i.e., “study population”), which involved demographic data and location, was considered to be indicative of good quality. All studies met criteria 8 (i.e., “levels of exposures”), 9 (i.e., “exposure measurement”), 11 (i.e., “outcome measurement”), and 12 (i.e., “blindness”). For the 14th criterion, a model was deemed sufficient if it controlled for sex (not stratified), age (not within ± 1 year), and a third confounder (e.g., socioeconomic status or academic performance). The articles were classified as “good” if they met at least 8 criteria, “fair” if they met between 5 and 7 criteria, and “poor” if they met fewer than 5 criteria.

**Table 2:** Checklist criteria, from the U.S. Department of Health & Human Services

1	Research objective: Was the research question or objective in this paper clearly stated?
2	Study population: Was the study population clearly specified and defined?
3	Participation rate ≥ 50%: Was the participation rate of eligible persons at least 50%?
4	Recruitment: Were all the subjects selected or recruited from the same or similar populations (including the same time period)? Were inclusion and exclusion criteria for being in the study prespecified and applied uniformly to all participants?
5	Sample size: Was a sample size justification, power description, or variance and effect estimates provided?
8	Levels of exposures: For exposures that can vary in amount or level, did the study examine different levels of the exposure as related to the outcome (e.g., categories of exposure, or exposure measured as continuous variable)?
9	Exposure measurement: Were the exposure measures (independent variables) clearly defined, valid, reliable, and implemented consistently across all study participants?
11	Outcome measurement: Were the outcome measures (dependent variables) clearly defined, valid, reliable, and implemented consistently across all study participants?
12	Blindness: Were the outcome assessors blinded to the exposure status of participants?
14	Confounding: Were key potential confounding variables measured and adjusted statistically for their impact on the relationship between exposure(s) and outcome(s)?

### Data Analysis

For the systematic review, a descriptive narrative analysis was conducted on 102 articles. For the meta-analysis, 52 articles were included, focusing on psychological correlates supported by at least three independent studies within each specific PLB domain. These correlates were initially categorized into three main theoretical areas: individual psychological factors, psychosocial factors, and partner-related factors. A subgroup meta-analysis was performed. Each psychological correlate of the PLB domain was treated as a distinct outcome, and a separate meta-analysis was conducted for each outcome. Thus, the current synthesis comprised six meta-analyses for ML, eleven for ED, and two for LA, totaling 19.

For the systematic review, a descriptive narrative analysis was conducted on 102 articles. For the meta-analytic synthesis, 50 articles were included, focusing on psychological correlates supported by at least three independent studies within each specific PLB domain. The synthesis was organized into three primary meta-analyses corresponding to the three PLB domains (ML, ED and LA). Within each domain, individual psychological correlates were treated as distinct outcomes. Consequently, the analysis examined five correlates for ML, eleven for ED, and two for LA, analyzing a total of 19 specific associations.

In our meta-analysis, the zero-order correlation coefficients (*r*) from the included studies that measured the relationship between PLB and specific psychological variables were treated as effect sizes. A positive *r* value indicates that a higher level of PLB is associated with a higher level of the corresponding variable. In terms of the effect sizes of the correlates, *r* coefficients of 0.10, 0.30, and 0.50 refer to small, medium, and large effects, respectively (Cohen, 1992).

To evaluate the heterogeneity among the studies included in each analysis, three statistics were employed: Cochran's *Q*, *Tau*^*2*^*,* and *I*^*2*^ values. Cochran's Q statistic was used to determine if the observed differences between studies were greater than would be expected by chance. A *p*-value associated with a *Q* statistic less than 0.05 indicates significant heterogeneity (Cochran, 1954). The Tau2 statistic provides an estimate of the between-study variance, representing the true variability in effect sizes beyond the sampling error (Borenstein et al., 2009). Finally, the I2 statistic describes the percentage of variability in effect sizes that is due to heterogeneity between studies rather than chance, with *I*^*2*^ values of 25%, 50%, and 75% indicating low, moderate, and high heterogeneity, respectively (Higgins et al., 2003).

A potential publication bias analysis was also performed. In meta-analysis research literature, the “file-drawer problem” phenomenon assumes that studies reporting statistically significant results are more likely to be published than those with non-significant findings (Rosenthal, 1979). If this assumption holds, it may lead to an estimation error in the true effect size calculation. To address this concern, several widely accepted methods in the meta-analysis literature were employed to assess the likelihood of publication bias. First, a funnel plot was used to estimate the presence or absence of publication bias. If the effect sizes of the studies are symmetrically distributed around the mean effect size, this indicates the absence of publication bias (Borenstein et al., 2009). Additionally, the trim-and-fill method was used to further assess the potential for publication bias, following the approach proposed by Fernández-Castilla et al. (2021).

Finally, a moderation analysis was conducted for any meta-analysis that showed high heterogeneity (*I*^*2*^ > 50%). Specifically, the percentage of female participants in each sample (treated as a continuous variable) was examined as a potential moderator, addressing a theoretical gap regarding gender/sex differences in the PLB research. This analysis was performed using meta-regression, with the *β* coefficient indicating the expected change in the correlation coefficient (Fisher’s *z*-transformed *r*) for each unit increase in the moderator variable.

All statistical analyses were conducted using the meta-analysis module of the Jamovi platform (Jamovi Project, 2025).

## Results

The characteristics of the 102 articles included in this systematic review are summarized in Table S1.

### Study Description (Sample, Conceptualization, and Assessment Tools)

Considering the articles included in the systematic review, the conceptual domains were distributed as follows: manic love (ML, *n* = 55, 53.9%), emotional dependence (*n* = 34, 33.3%), and love addiction (*n* = 13, 12.7%). The study design was cross-sectional for all cases (*n* = 102, 100%). The samples were collected from Europe (*n* = 54, 52.9%), the United States (*n* = 23, 22.5%), Latin America (*n* = 12, 11.8%), and Asia (*n* = 9, 0.9%). Four studies included samples from different countries. Specifically, these studies were conducted in Turkey and the UK (Study 82); the UK and Hong Kong (Study 86); the US, Mozambique, and Portugal (Study 18); and Australia, Canada, New Zealand, America, Germany, the United Kingdom, Italy, the Netherlands, Puerto Rico, Turkey, and the United Arab Emirates (Study 46).

Studies on ML were predominantly conducted in the US (*n* = 23), with the remainder conducted in the UK (*n* = 4), Portugal (*n* = 5), Hungary (*n* = 2), Spain (*n* = 2), Switzerland (*n* = 2), Poland (*n* = 1), Brazil (*n* = 1), Serbia (*n* = 2), Malaysia (*n* = 1), Italy (*n* = 1), Turkey (*n* = 1), Mexico (*n* = 2), Israel (*n* = 1), Japan (*n* = 1), Palestine (*n* = 1), and Germany (*n* = 1). Studies on ED were mainly conducted in Spain (*n* = 20), with the remainder conducted in Ecuador (*n* = 3), Turkey (*n* = 3), Italy (*n* = 2), Netherlands (*n* = 1), Brazil (*n* = 1), Mexico (*n* = 1), and Peru (*n* = 3). Most studies on LA were conducted in Italy (*n* = 9), except for one from Turkey (Study 3), one from the UK (Study 24), one from Brazil (Study 102), and one from China (Study 96).

Regarding the year of publication, the included articles spanned 1993 to 2025. However, in the case of love addiction, the research began in 2021. The sample size ranged from 63 (Study 83) to 3,375 (Study 77). The percentage of women in the sample ranged from 0% (Study 83) to 94.2% (Study 17).

Eleven evaluation instruments were used (Table 3). High scores indicate a high degree of PLB. The Mania subscale of the Love Attitudes Scale (LAS; Hendrick & Hendrick, 1986), in its various versions, was the most commonly used instrument. Additionally, in the evaluation of emotional dependence, the Emotional Dependence Questionnaire (Lemos & Londoño, 2006) was the most prevalent, and in the case of love addiction, the Love Addiction Inventory (LAI) (Costa et al., 2021) was the most prevalent.

**Table 3:** Assessment tools

Instruments	Version	Studies^a^
Addiction in Romantic Relationships Scale (Atlam et al., 2023)	Original—Turkish (Atlam et al., 2023)	3
Emotional Dependence in Dating scale (DEN, Urbiola et al., 2014a, 2014b)	Original—Spanish (Urbiola et al., 2014a, 2014b)	30
Emotional Dependence Inventory (IDE, Inventario de Dependencia Emocional, Aiquipa, 2012)	Original—Spanish (Aiquipa, 2012)	1
Emotional Dependence Questionnaire (Cuestionario de Dependencia Emocional, CDE; Lemos & Londoño, 2006)	Original—Spanish (Lemos & Londoño, 2006)	4, 5, 13, 15, 16, 29, 31, 44, 54, 64, 67, 75, 76, 80, 92, 93
	Brazilian version (Fonsêca et al., 2020)	22
Emotional Dependency Scale (EDS) (Buunk, 1981)	Original—Turkish (Buunk, 1981)	50, 84, 89, 100
Emotional Dependence Scale (Jiménez-Cruz et al., 2022)	Original—Spanish (Jiménez-Cruz et al., 2022)	45
Interpersonal Relationships and Emotional Dependencies Inventory (IRIDS-100, Inventario de Relaciones Interpersonales y Dependencias Sentimentales, Ruiz & de la Villa Moral Jiménez, 2018). Affective Dependence Subscale	Original—Spanish (Ruiz & de la Villa Moral Jiménez, 2018)	10, 20, 21, 26, 41, 65, 66, 68
Love Addiction Inventory (LAI) (Costa et al., 2021)	Original—Italian (Costa et al., 2021)	8, 11, 14, 17, 35, 96
	Short-Form—Italian (Costa et al., 2021)	24, 38, 39, 40, 90
	Original—Brazilian version (Zibenberg & Natividade, 2025)	102
Love Attitudes Scale (LAS) (Hendrick & Hendrick, 1986). Mania subscale	Original—English (Hendrick & Hendrick, 1986)	2, 6, 7, 9, 19, 34, 36, 43, 46, 47, 48, 51, 53, 61, 62, 63, 69, 74, 79^#^, 83, 91^#^, 99, 101
	Short-Form – English (LAS-SF; Hendrick et al., 1998)	18, 28, 32, 36, 42, 56^#^,57, 58, 59, 82, 85, 86, 88, 98
	Short-Form – Italian version (Agus et al., 2018)	55
	Portuguese version (Neto, 1994)	12, 18, 70, 71, 72, 73, 87
	Spanish version (Arias-Galicia, 1989)	23
	Japanese version (Matsui et al., 1990)	49
	Short-Form – Turkish version (Buyuksahin, 2005)	27 82
	Short-Form – Hungarian version (Meskó et al., 2021)	52 60
	Serbian version (Dinić & Jovanović, 2021)	25
	Short-form – Malaysian version (Wan Shahrazad et al., 2012)	97
	Short-form – Spanish version (Rodríguez-Castro et al., 2013)	34
Marburg Attitude Inventory for Love Styles (MEIL; Bierhoff et al., 1993)	Original – German (Bierhoff et al., 1993)	78 81 94
Spouse-Specific Dependency Scale (Rathus & O'Leary, 1997). Emotional Dependency subscale	Original—English (Rathus & O'Leary, 1997)	77
	Spanish version (Valor-Segura, Exposito & Moya, 2008)	95

*Note*: ^a^ID number assigned in Table S1 (Supplemental Material). ^#^ Translated by authors

In the quality evaluation (Table S1), 24 studies (23.5%) were classified as good quality, 78 (76.5%) as fair quality, and none as poor quality. The most frequent limitations were the lack of a complete definition of the population, specifically the timing of sample recruitment (Criterion 2) (*n* = 12; 11.8%), failure to report the participation rates of eligible persons (Criterion 3) (n = 80; 78.4%), and the lack of power analysis or provision of variance and effect size estimates to justify the sample size (Criterion 5) (*n* = 89; 87.2%). Furthermore, 101 studies (99.0%) did not account for confounding variables or adjust for their influence on the relationship between exposure and outcome, as they only used bivariate correlation analyses (Criterion 14). The sole exception was the Study 14, who reported only partial correlations.

### Factors Correlated with Problematic Love Behaviors

#### Manic Love

##### Individual Psychological Factors

Focusing on the Big Five personality traits, some studies have found significant positive correlations with neuroticism (Study 98). Other studies have found associations with temperamental factors such as emotionality and impulsivity (Study 19) and distress and fearfulness (Study 99). Variables related to self-representation, such as low self-esteem (Studies 9, 19, 61, 101, 102) and self-esteem instability (Study 101), were also significantly associated. Additional factors include loneliness (Study 61), shyness, and anger self-disclosure (Study 28). Variables related to self-representations, such as high private self-consciousness, public self-consciousness, social anxiety, and low public performance (Study 72), as well as individual-level values, including low conservation and low openness to change (Study 82), were also significantly associated.

ML were consistently associated with pathological personality traits, including narcissism, vulnerable narcissism (Studies 6 and 81), Machiavellianism (Study 46), and traits of the Dark Triad, such as antagonism, psychoticism, detachment, negative affectivity, and disinhibition (Studies 47 and 61). Significant relationships have also been reported with psychopathy (Study 6) and a range of disorders, including depression, anxiety, alcohol dependence, and borderline personality disorder (Study 2), narcissism (Studies 9, 25 and 62), as well as psychopathy (Study 6). Additional associations were found with depression, major depression, alcohol dependence, disclosure, borderline personality disorder, passive-aggressive behavior, self-defeating behavior, avoidant personality disorder, dysthymia, somatoform disorders, anxiety, and schizotypal personality disorder (Study 2). Conversely, ML has been found to be negatively associated with borderline and histrionic personality traits specifically among females (Study 88).

Significant relationships have also been found with sexual attitudes of communion (Study 96) and low sociosexual orientation (Study 60). Additionally, associations have been found with reasons for having sex, including personal goal attainment, relational reasons, sex as coping (Study 60), socio-sexual desire (Study 56), and low socio-sexuality (Study 73). Study 71 found positive associations between pathological love, sexual desire, and commitment.

Other related attitudes include the favorability of attitudes toward one's favorite celebrity (Studies 58 and 59) and low attitudes towards pets (Study 42). Additionally, it has been linked to low well-being (Studies 18 and 85) and low relationship satisfaction (in general, not current) (Study 18).

##### Psychosocial Factors

ML is negatively associated with secure attachment and positively associated with insecure anxiety (Studies 57 and 58). However, one study reported a negative relationship between ML and both types of insecure attachment (Study 86).

##### Partner-Related Factors

ML was significantly associated with attitudes towards polyamory (Study 32), romanticism (Study 48), low capacity to love (Study 6), jealousy (Studies 28, 37, 57 and 91), passion and low intimacy (Study 12), romanticism, low sensation, and low conformity as ways to be compatible (Study 55), and the soulmate theory of relationships (Study 58). One study found that pathological love was associated with sensitivity as an attractive partner characteristic and was negatively associated with ratings of intelligence and a partner being good in bed. As an intensification strategy, pathological love was correlated with tokens of affection, enhancement of physical appearance, and behavioral adaptation. It is also associated with ratings on several tests, including triangle, separation, endurance, and indirect tests (Study 53).

Some studies have shown significant associations between ML and low relationship satisfaction (Studies 7, 12, 27, 42, and 87).

ML has been linked to domination, submission, and interactive reactivity (Study 59). Study 34 further highlighted these dynamics, finding a negative association between pathological love and emotional clarity and repair, and a positive association with emotional flooding, conflict frequency, and conflict intensity. Study 49 found that, in both males and females, ML is associated with emotional experiences of anxiety/jealousy, liveliness, affection, and envy. Also, in female populations, it is also significantly linked to feelings of uncertainty.

Study 51 found positive correlations between ML and communication values, specifically affectively oriented skills such as conflict management, comforting, ego support, and regulative skills, as well as instrumentally oriented skills such as persuasive, conversational, and referential skills. Study 36 found significant positive correlations between ML and negative relational maintenance behaviors such as jealousy induction, avoidance, spying, infidelity, destructive conflict, and allowing control. They also found negative correlations between ML and relational quality indicators, such as satisfaction, control mutuality, and respect. Study 23 found significant positive correlations between ML and emotional negotiation.

ML has been linked to both general intimate partner violence (Study 79), aggression (Study 7), violence perpetrated and received (Study 33), and minor psychological aggression perpetrated (Study 23). Conversely, ML has been found to be negatively associated with nonverbal sexual arousal perpetration, emotional manipulation and deception perpetration, and nonverbal sexual arousal victimization among females (Study 88).

#### Emotional Dependence

##### Individual Psychological Factors

Focusing on the Big Five personality traits, some studies have found significant correlations with neuroticism (Study 22) and low extraversion (Study 22). Additionally, relationships have been identified with locus of control variables, including internality, chance externality, and powerful others externality (Study 22). Other personality variables that show significant relationships include intolerance of uncertainty, inhibition-generating uncertainty, uncertainty as bewilderment and unpredictability, tendency to worry, and pessimism (Study 68), low self-esteem (Studies 29, 76, and 93), and loneliness (Study 20). Additionally, significant relationships have been found with impulsivity (Studies 30 and 76).

In terms of psychopathology, associations have been found with depression and anxiety (Studies 10 and 29), social anxiety and fear of negative evaluation (Study 67), general psychopathology (Study 10), and difficulties in emotion regulation (Study 31). Study 20 also found positive relationships with a cluster of negative feelings and experiences, including loneliness, negative feelings, emptiness, self-destruction, inescapability, and guilt, among others.

Some of the included studies linked emotional dependence to alcohol and substance use/abuse (Studies 4, 22, and 65). It has also been associated with behavioral addictions such as exercise addiction (Studies 75 and 76), Internet addiction (Study 29 and 22), smartphone addiction (Study 29), social media use/addiction (Studies 26 and 64), gambling addiction, eating disorders, gaming addiction, compulsive spending, and sex addiction (Study 22).

Additionally, emotional dependence has been linked to low resilience (Study 66), low perceived social support (Study 45), and low life satisfaction (Study 1). However, Study 96 found positive relationships with four protective factors that confer resilience: social support, social skills, goal efficacy, and planning behavior.

##### Psychosocial Factors

Emotional dependence is associated with early maladaptive schemas, such as emotional deprivation, abandonment, subjugation, mistrust, failure, dependence, enmeshment, emotional inhibition, unrelenting standards, entitlement, insufficient self-control, defectiveness, self-sacrifice, and attachment (Studies 31 and 92). There is also a connection between emotional dependence and childhood trauma (Studies 4, 65, and 75), including physical and emotional abuse (Study 65). Other related factors include parental interference, regard for authority, self-sufficiency, and resentment toward parents (Study 75).

Emotional dependence is positively associated with insecure anxiety (Studies 4 and 65), avoidant attachment (Studies 5 and 65), and disorganized attachment (Study 65). Other studies have found a negative relationship between emotional dependence and these two types of insecure attachment (Study 10).

##### Partner-Related Factors

Emotional dependence is significantly associated with forgiveness (Study 89), interpersonal misperception, unrealistic relationship expectations (Study 50), separation anxiety, partner's affective expression, changing plans, fear of loneliness, borderline expression, attention-seeking (Study 75), intimacy and low autonomy (Study 84), need for exclusivity, avoidance of being alone, need to please, asymmetry (Study 29), and relationship beliefs (Study 100).

A study found that emotional dependence was associated with relationship satisfaction as well as with relationship adjustment (Study 89). This study also found a negative association between emotional dependence and conflict resolution responses, such as exit and neglect responses.

Emotional dependence is associated with both general intimate partner violence (Studies 1, 41, 44, and 45) and violence perpetrated and received (Studies 13 and 76). In terms of violence perpetration, significant relationships were found between psychological abuse, physical aggression, sexual coercion, and low injury (Study 80), social psychological violence, control, and humiliation (Study 93), perpetrated cyber control (Study 21), and direct and control perpetuation (Study 95). Violence received has been associated with psychological violence (Studies 21, 22, 41, 65, 66, 67, 80, 93, and 95), physical (Studies 22, 67, and 80), and sexual (Study 22 and 80).

#### Love Addiction

##### Individual Psychological Factors

Focusing on the Big Five personality traits, LA correlated significantly with neuroticism and low scores on agreeableness, openness, and conscientiousness (Study 39). It is also associated with low self-esteem (Studies 38 and 102). Additionally, significant relationships were found with subdimensions of impulsive behavior, such as positive urgency, negative urgency, and sensation seeking (Study 24). Furthermore, LA has been linked to vulnerable narcissism (Study 11).

In terms of psychopathology, associations have been found with depression and anxiety (Study 35), negative affect (Study 17), difficulties in emotion regulation (Study 24), and coping strategies (Study 35). It has also been associated with social media addiction (Studies 35 and 39), problematic online dating app use, cyber pornography addiction (Study 39), and hypersexual behavior (Study 8). Additionally, it has been linked to low resilience (Study 35) and low positive affect (Study 17). Moreover, a study found significant associations between love addiction and neurotic and immature defense mechanisms (Study 90).

Study 35 found significant positive relationships between perceived cognitive, memory, and attentional failures and cognitive function at work, and a negative relationship with the frequency of memory failures.

##### Psychosocial Factors

LA has been linked to family functioning, specifically low cohesion and high levels of disengagement, enmeshment, rigidity, and chaotic environments (Study 40). There is also a connection between LA and childhood trauma, including physical and emotional abuse (Study 40). Other related factors include low parental care and protection (Study 3). Additionally, LA was positively associated with insecure anxiety attachment to parents (Atlam et al., 2023).

##### Partner-Related Factors

Study 14 found significant positive correlations between love addiction and anxious adult attachment. The study also revealed positive associations between the total score and all subscales of Relational Obsessive–Compulsive Disorder, including obsessions related to love for the partner, relationship adequacy, and the partner's love. Additionally, focusing on emotional dynamics, the authors found a positive correlation between love addiction and a lack of dyadic clarity, a subscale of difficulties in emotion regulation in the dyadic context. The study also found that love addiction was positively correlated with the tendency to enter tolerance-battering relationships, specifically linking this pattern to insecure attachment, underserving self-image, and a self-sacrificing nature.

Additionally, LA is positively associated with insecure attachments to partners (Studies 38 and 90). In line with these negative relational patterns, Study 96 found that love addiction had a positive relationship with the sense of giving and perceived acceptability of gaslighting and a negative relationship with relationship power. Furthermore, gender-specific associations have been identified, with LA being linked to emotional abuse, as well as emotional and physical neglect, exclusively among female populations (Study 11).

### Meta-Analysis

#### Manic Love

A total of 25 studies involving five correlates met the inclusion criteria, and 49 effect sizes were obtained in this meta-analysis. The results of the overall analysis of the relationship between love addiction and psychological outcomes are presented in Table 4. In the Supplementary Material (Fig. 1S–6S), each forest plot corresponding to each factor can be consulted.

**Table 4:** Results for the meta-analysis of relationship between manic love and individual psychological factors, psychosocial factors, and partner-related factors

Type of correlate	No. studies	No. ES	Mean r (SD)	95% CI	z value (sig)	Q (sig)	Tau^2^	I^2^ (%)	Publication bias^a^
*Individual psychological factors*									
Self-esteem	4	4	− 0.34 (0.04)	(− 0.40, − 0.27)	− 9.45***	2.78	0.00	8.72	0
Narcissism	7	13	0.26 (0.05)	(0.15, 0.36)	4.86***	94.27***	0.03	89.39	0
Psychopathy	3	4	0.24 (0.09)	(0.06, 0.41)	2.70**	34.12***	0.03	91.98	1
*Partner-related factors*									
Jealousy	4	8	0.35 (0.05)	(0.23, 0.45)	6.35***	36.97***	0.02	85.03	0
Violence perpetrated	3	10	− 0.02 (0.05)	(− 0.12, 0.07)	− 0.52	33.22***	0.02	71.87	2
Relationship satisfaction	8	10	− 0.15 (0.03)	(− 0.21, − 0.09)	− 4.94***	30.71***	0.01	72.76	0

^a^Number of missing studies estimated by the trim-and-fill method (L0 +)

**p < .01, ***p < .001

Of the six correlates analyzed, five were significantly associated with ML. Specifically, three were positively associated: narcissism (*r* = .26, *p* < .001), psychopathy (*r* = .24, *p* < .01), and jealousy (*r* = .35, *p* < .001). Conversely, self-esteem (*r* = − .34, *p* < .001) and relationship satisfaction (*r* = − .15, *p* < .001) were negatively associated with ML. The effect sizes were small for relationship satisfaction, psychopathy, and narcissism, while while jealousy and self-esteem reached medium effect sizes.

Regarding the heterogeneity indices, the *Tau*^*2*^ values were low (< .10), indicating a relatively small amount of between-study variance. However, the Q statistic was significant for four correlates, including narcissism, psychopathy, jealousy, and relationship satisfaction, indicating statistically significant heterogeneity. The *I*^*2*^ values were all above 75%, except for self-esteem, violence perpetrated and relationship satisfaction, suggesting substantial heterogeneity.

Regarding publication bias, as shown in the Supplemental Material (Fig. 19S–25S), the funnel plots for the five correlates were generally symmetrically distributed, suggesting minimal evidence of publication bias. Additionally, as suggested by Fernández-Castilla et al. (2021) for the trim-and-fill method, the optimal values for detecting publication bias would vary based on the magnitude of effect sizes (*r* = .14 to .35 in this study) and the number of effect sizes (4 to 13 in this study) included in the analysis. Under these conditions, publication bias was indicated if L0 + was greater than 2. Our results from the trim-and-fill method for all correlates showed L0 +  = 0, except for psychopathy (L0 +  = 1) and violence perpetrated (L0 +  = 2), indicating no substantial evidence of publication bias.

#### Emotional Dependence

A total of 20 studies involving 10 correlates met the inclusion criteria, and 30 effect sizes were obtained in the current meta-analysis. The results of the overall analysis of the relationship between emotional dependence and psychological outcomes are presented in Table 5. In the Supplementary Material (Fig. 7S–16S), each forest plot corresponding to each factor can be consulted.

**Table 5:** Results for the meta-analysis of relationship between emotional dependence and individual psychological factors, psychosocial factors, and partner-related factors

Type of correlate	No. studies	No. ES	Mean r (SD)	95% CI	z value (sig)	Q (sig)	Tau^2^	I^2^ (%)	Publication bias^a^
*Individual psychological factors*									
Self-esteem	3	3	− 0.31 (0.02)	(− 0.35, − 0.26)	− 13.7***	0.23	0.00	0	0
Alcohol use/abuse	4	6	0.16 (0.02)	(0.12, 0.20)	7.89***	10.29	0.00	54.07	3
Use/abuse of other substances	4	14	0.10 (0.01)	(0.08, 0.11)	13.4***	15.30	0.00	0.28	0
Behavioral addictions	5	10	0.19 (0.02)	(0.15, 0.23)	10.1***	31.28	0.00	75.75	0
*Psychosocial factors*									
Anxious attachment	4	6	0.15 (0.12)	(− 0.08, 0.37)	1.26	234.68***	0.08	97.79	0
Avoidant attachment	3	3	0.24 (0.05)	(0.14, 0.34)	4.62	4.91***	0.00	62.28	0
*Partner-related factors*									
Psychological violence received	8	16	0.34 (0.04)	(0.26, 0.43)	8.07***	313.66***	0.03	95.49	0
Physical violence received	3	3	0.29 (0.12)	(0.05, 0.52)	2.34***	44.22***	0.04	96.72	0
Violence perpetrated	6	12	0.22 (0.04)	(0.15, 0.29)	5.80***	156.13***	0.02	94.25	0
Relationship satisfaction	3	3	0.42 (0.20)	(0.03, 0.81)	2.14*	85.94***	0.11	97.42	0

^a^Number of missing studies estimated by the trim-and-fill method (L0 +)

*p < .05, ***p < .001

Of the 10 correlates analyzed, eight were significantly associated with emotional dependence. Specifically, eight were positively associated: alcohol use/abuse (*r* = .16, *p* < .001), use/abuse of other substances (*r* = .10, *p* < .001), behavioral addictions (*r* = .19, *p* < .001), psychological violence received (*r* = .34, *p* < .001), physical violence received (*r* = .29, *p* < .001), violence perpetrated (*r* = .22, *p* < .001), and relationship satisfaction (*r* = .42, *p* < .05). Self-esteem was negatively associated with emotional dependence *(r* = -.31, *p* < .001). The effect sizes were small in all cases except for psychological violence received, relationship satisfaction, and self-esteem, which were medium.

Regarding heterogeneity indices, the *Tau*^*2*^ values were low (< .10) for most correlates, except for relationship satisfaction, suggesting a relatively small between-study variance. However, seven correlates, excluding relationship satisfaction, showed a significant *Q* statistic, indicating a statistically significant heterogeneity. The *I*^*2*^ values were all above 75%, except for self-esteem, use/abuse of other substances, and avoidant attachment, further suggesting substantial heterogeneity among studies.

Regarding publication bias, as shown in the Supplemental Material (Fig. 25S–33S), the funnel plots for the 11 correlates were generally symmetrically distributed, suggesting minimal evidence of publication bias. Additionally, as suggested by Fernández-Castilla et al. (2021) for the trim-and-fill method, the optimal values for detecting publication bias would vary based on the magnitude of effect sizes (*r* = .10 to .42 in this study) and the number of effect sizes (3 to 16 in this study) included in the analysis. Under these conditions, publication bias was indicated if L0 + was greater than 2. Our results from the trim-and-fill method for all correlates showed L0 +  = 0, except for alcohol use/abuse (L0 +  = 3). Therefore, substantial evidence of publication bias was only found for the correlation of alcohol use/abuse.

#### Love Addiction

Six studies involving two correlates met the inclusion criteria, and eight effect sizes were obtained in the current meta-analysis. The results of the overall analysis of the relationship between love addiction and psychological outcomes are presented in Table 6. In the Supplementary Material (Fig. 17S–18S), each forest plot corresponding to each factor can be consulted.

**Table 6:** Results for the meta-analysis of relationship between love addiction and individual psychological factors, psychosocial factors, and partner-related factors

Type of correlate	No. studies	No. ES	Mean r (SD)	95% CI	z value (sig)	Q (sig)	Tau^2^	I^2^ (%)	Publication bias^a^
*Individual psychological factors*									
Behavioral addictions	3	5	0.37 (0.02)	(0.32, 0.41)	17.1***	3.27	0	0	0
*Psychosocial factors*									
Anxious attachment	4	6	0.34 (0.09)	(0.16, 0.52)	3.75***	146.01***	0.05	95.65	0

^a^Number of missing studies estimated by the trim-and-fill method (L0 +)

***p < .001

Both correlates showed a significant and positive association with LA: behavioral addictions (*r* = .37, *p* < .001) had a medium effect size, while anxious attachment (*r* = .34, *p* < .001) showed a strong effect size.

Regarding heterogeneity indices, the *Tau*^*2*^ value was low (< .10), suggesting a relatively small between-study variance. However, anxious attachment showed a significant *Q* statistic, indicating a statistically significant heterogeneity. The corresponding *I*^*2*^ value was above 75%, further suggesting substantial heterogeneity.

Regarding publication bias, as shown in the Supplemental Material (Fig. 27S–28S), the funnel plots for the two correlates were generally symmetrically distributed, suggesting minimal evidence of publication bias. Additionally, as suggested by Fernández-Castilla et al. (2021) for the trim-and-fill method, the optimal values for detecting publication bias would vary based on the magnitude of effect sizes (*r* = .34 to .37 in this study) and the number of effect sizes (5 to 6 in this study) included in the analysis. Under these conditions, publication bias was indicated if L0 + was greater than 2. Our results from the trim-and-fill method for the two correlates showed L0 +  = 0, which indicated no substantial evidence of publication bias.

### Moderation by Percentage of Female Participants

A moderation analysis was conducted to examine the influence of the percentage of female participants in the sample as a moderator of the effect sizes for those significant correlates that exhibited moderate to high residual heterogeneity (*I*^*2*^ > 50%) and included at least four effect sizes. This analysis revealed a significant moderating effect of sample composition on two specific correlates, partially accounting for the observed heterogeneity. The complete results of this analysis are presented in Table 7.

**Table 7:** Meta-regression: Moderation by percentage of female participants

Variables	β (SD)	95% CI	z value (sig)
*Manic love (ML)*			
Narcissism	− 0.53 (0.61)	(− 1.71, 0.65)	− 0.88
Psychopathy	− 0.10 (0.68)	(− 1.42, 1.24)	− 0.13
Jealousy	1.28 (0.31)	(0.68, 1.88)	4.18***
Violence perpetrated	− 0.04 (0.15)	(− 0.34, 0.26)	− 0.28
Relationship satisfaction	− 0.48 (0.16)	(− 0.80, − 0.17)	− 3.01**
*Emotional dependence (ED)*			
Alcohol use/abuse	− 0.01 (0.24)	(− 0.47, 0.45)	− 0.05
Behavioral addictions	0.21 (0.12)	(− 0.01, 0.44)	1.84
Psychological violence received	− 0.18 (0.33)	(− 0.84, 0.47)	− 0.55
Violence perpetrated	− 0.17 (0.44)	(− 1.03, 0.69)	− 0.38
Relationship satisfaction	0.06 (0.04)	(− 0.01, 0.13)	1.74
*Love addiction (LA)*			
Anxious attachment	− 2.63	(− 3.72, − 1.73)	− 5.74***

For ML, significant positive moderating effects were found in the correlation with jealousy (*β* = 1.28, *p* < 0.001) and relationship satisfaction ((*β* = -0.48, *p* < 0.01). This finding indicates These results indicate that the positive association between ML and jealousy becomes significantly stronger as the proportion of women in the sample increases. Similarly, the negative association between ML and relationship satisfaction becomes more pronounced in samples with a higher percentage of female participants.

Regarding ED, none of the correlates analyzed showed a significant moderating effect as a function of the percentage of female participants.

Finally, in the LA domain, a significant negative moderating effect was observed for the correlation with anxious attachment (*β* = -2.63, *p* < 0.001). This negative coefficient indicates that the association between LA and Anxious Attachment was stronger in studies with a lower proportion of female participants.

## Discussion

In some instances, romantic relationships can become unhealthy and are associated with negative psychosocial outcomes. Some existing studies have investigated the factors associated with these problematic forms of love. This study aimed to address the high conceptual and statistical heterogeneity in the field through a systematic review and subgroup meta-analyses. Specifically, this study sought to: (1) examine the distinct conceptualizations and assessment instruments used for manic love, emotional dependence, and love addiction; (2) review the specific correlates across these three domains; (3) perform subgroup meta-analyses of established correlates; and (4) investigate the role of gender/sex as a potential moderating factor in the magnitude of the correlates' effect sizes.

Regarding Objective 1, most of the included studies (53.9%) examined Lee's (1973) concept of manic love. Additionally, a significant portion (33.3%) analyses the concept of emotional dependence while a smaller fraction (12.7%) studies the term 'addiction.' Furthermore, the study of these concepts appears to be influenced by the country of origin. Specifically, most studies investigating mania originate from the US, those focusing on emotional dependence come from Spain or Spanish-speaking countries, and those examining love addiction come from Italy. This finding may be related to cultural differences in the conceptualization of love (Heshmati et al., 2019), which may similarly influence the understanding of problematic love. This may be due to the availability of validated assessment instruments in different languages.

Indeed, it has been found that the most used instruments for evaluating manic love are the mania subscale of the ‘Love Attitudes Scale’ (LAS, Hendrick & Hendrick, 1986; Hendrick et al., 1998). This subscale comprises seven items in its original version (Hendrick & Hendrick, 1986) and four items in its short form (Hendrick et al., 1998), which refer to obsessive and dependent love attitudes (e.g., “When my lover doesn't pay attention to me, I feel sick all over”), based on Lee's (1973) theory. As observed in the results, this scale has been adapted and validated in multiple languages, including Japanese (Matsui et al., 1990), Portuguese (Neto, 1994), Italian (Agus et al., 2018), Turkish (Buyuksahin, 2005), Hungarian (Meskó et al., 2021), Serbian (Dinić & Jovanović, 2021), Spanish (Rodríguez-Castro et al., 2013), and Malaysian (Wan Shahrazad et al., 2012).

The Emotional Dependence Questionnaire (Lemos & Londoño, 2006) was the most frequently used tool for evaluating emotional dependence. This instrument comprises 23 items and six components that address separation anxiety, affective expression from the partner, modification of plans, fear of loneliness, extreme reactions to potential loss, and seeking the partner’s attention. This instrument was based on Castelló's (2005) theory of emotional dependency. According to this theory, individuals with high emotional dependency are characterized by the submission and idealization of their partner, low self-esteem, and an overwhelming need for the other person, leading to clingy behaviors and a profound fear of loneliness. The Love Addiction Inventory (LAI, Costa et al., 2021) is a widely used tool for assessing love addiction. This instrument, with a long version of 24 items and a short version of six items, uses the six elements of addiction (Griffiths, 2005). Thus, it can be stated that these three instruments assess problematic aspects of couple relationships based on lack of control, mood modification, obsession, prioritization, and dependence. However, they differ in the theoretical models that support these assumptions.

Regarding Objective 2, the systematic review revealed a wide range of factors correlated with the three PLBs, which were categorized into three main groups: individual psychological, psychosocial, and relationship-related factors. It is crucial to note that this synthesis was conducted on a primary literature base consisting almost entirely of cross-sectional studies that largely failed to control for confounding variables, thus yielding correlations that must be interpreted with caution. Thus, the broad range of correlates identified must be interpreted with caution, as the correlations presented in the existing literature are predominantly bivariate and not adjusted for the influence of key variables such as sociodemographic (e.g., age) or psychological factors (e.g., personality traits, general psychopathology, or trauma history).

Regarding Objective 3, the results of the subgroup meta-analyses indicated that among the previous factors, several individual, psychosocial, and partner-related factors showed significant correlations, supported by evidence from at least three studies within each domain. In the case of manic love, the significant correlates were limited to five specific factors: low self-esteem, narcissism, psychopathy, jealousy and low relationship satisfaction. On the other hand, emotional dependence exhibited the broadest range of correlates, including low self-esteem, alcohol use/abuse, other substance use/abuse, behavioral addictions, psychological violence received, physical violence received, perpetrated violence, and relationship satisfaction. Finally, the significant correlates of love addiction were behavioral addictions and anxious insecure attachment.

Focusing on shared factors, low self-esteem was a significant common correlate shared by manic love and emotional dependence, suggesting a core vulnerability across these two domains. Furthermore, behavioral addictions were found to be common to emotional dependence and love addiction, reinforcing the notion that both share characteristics with addictive disorders (Camarillo et al., 2020; Costa et al., 2021).

Focusing on divergences, relationship satisfaction exhibited opposite patterns of association across the PLB domains: while it was significantly and positively associated with emotional dependence, a significant negative association was found with manic love attitudes. Furthermore, anxious insecure attachment was significantly associated with love addiction but not a significant meta-analytic correlate of emotional dependence. These differential findings provide strong empirical support for our overarching hypothesis that these three PLBs, while related, are distinct behavioral phenomena that must be studied separately to achieve etiological clarity and avoid conceptual confounding.

Finally, in relation to Objective 4, significant moderations were identified between manic love and jealousy, manic love and relationship satisfaction, and love addiction and anxious attachment, which contributed to reducing the statistical heterogeneity observed in the meta-analyses for these two variables. The finding of a stronger, more positive correlation between manic love and jealousy in female-predominant samples aligns with previous evidence suggesting that, in females, negative relational dynamics are more often linked to negative emotions, such as jealousy, whereas in males, they are more associated with aggressive and externalizing behaviors (Banaszkiewicz, 2022). Similarly, the inverse relationship between manic love and relationship satisfaction was more pronounced in female-predominant samples, suggesting that women may experience the emotional toll of manic love attitudes more acutely. Conversely, the moderation in the correlation between love addiction and anxious attachment was negative, indicating that this association was stronger in studies with a lower proportion of women (i.e., male-predominant samples). This result differs from the findings of a recent meta-analysis by Cavalli et al. (2025), who did not find significant gender/sex moderation. This discrepancy is likely attributable to the methodological differences used in the present study, which analyzed each PLB domain separately, thereby allowing these specific subgroup effects to emerge.

Regarding the limitations of the included studies, none met the quality criterion for controlling confounding variables (Criterion 14), as the reported correlations were bivariate rather than partial or adjusted. This analytical flaw in 99.0% of the primary literature means that the observed correlations may be spurious or inflated, as they have not been adjusted for the influence of key variables (e.g., general psychopathology, trauma, or socioeconomic status). Therefore, future research must employ robust multivariate designs to rigorously isolate the unique effects of specific correlates of PLBs. Furthermore, most studies did not report participation rates (Criterion 3), which may be because data collection often relies on online surveys, making it difficult to determine the number of people reached. Additionally, the sample sizes were not justified (Criterion 5), leaving uncertainty regarding their adequacy. Finally, although the studies provided descriptions of their samples (Criterion 2), they did not specify when data collection took place, preventing us from determining whether the studies were up-to-date.

Regarding the limitations of this study, it is important to first highlight that a critical constraint stems from the design of the primary literature. As the included studies were almost entirely cross-sectional, our meta-analysis could only establish correlational associations between PLBs and the identified psychological variables. Consequently, it was impossible to determine the directionality of these relationships. This fundamental design flaw in the evidence base means that our results cannot infer causality or predictive power. Therefore, future research must move beyond simple cross-sectional designs to utilize longitudinal studies, specifically employing advanced methods such as longitudinal designs, which are necessary to disentangle the temporal sequence and establish causal inferences for these complex problematic behaviors.

Second, the statistical properties of the meta-analysis are limited. Significantly high heterogeneity was observed in the meta-analyses for certain individual correlates. Moderation analysis (Objective 4) successfully explained a portion of this variance by identifying the percentage of female participants as a significant moderator in two key correlates (one for ML and one for LA). However, the underlying source of this heterogeneity remains unknown. This residual variance is likely attributable to the combined effects of the diverse assessment instruments used to measure PLBs or the sociocultural characteristics of the different samples (e.g., country of origin). Furthermore, the limited number of studies included in many of our individual meta-analyses restricted the possibility of conducting rigorous moderation analyses on unexamined variables. Since PLBs are currently an emerging topic in research, it is highly probable that in the future, with a greater number of empirical studies available, it will be possible to replicate and expand the findings of this study through more detailed moderation analyses, specifically focusing on reducing the residual heterogeneity for those correlates for which instruments or cultural differences are suspected to be influential.

Third, the potential for systematic bias introduced by linguistic restrictions is a limitation. The search was restricted to studies published in English, Spanish, French, and Italian because of the language proficiency of the research team. Restricting the search to these four languages may have introduced sampling bias by excluding significant portions of the global literature. Therefore, future systematic reviews should involve broader, multinational research teams to incorporate a wider array of languages and ensure a more comprehensive representation of global PLB research.

Fourth, it is important to acknowledge that the studies included in this review focused exclusively on dyadic romantic relationships. Consequently, our findings may not be generalizable to other relational structures, such as polyamorous relationships or other forms of consensual non-monogamy. The dynamics of emotional dependency, manic love, or love addiction may manifest differently in non-dyadic contexts, where attachment and commitment are negotiated across multiple partners. Future research should explore whether these problematic patterns and their correlates follow similar trajectories across diverse relational frameworks.

Finally, our decision to focus on non-clinical populations, while intentional to ensure methodological homogeneity and a focus on community-based prevention, limits the generalizability of our findings to clinical settings. Future research involving clinical samples could clarify whether the identified correlates operate differently at higher levels of severity or in the presence of formal psychiatric comorbidities.

### Conclusions

Despite these limitations, this study makes an important contribution to the field of problematic romantic relationships. To date, this is the first systematic review and subgroup meta-analysis of factors correlated with the three distinct PLB constructs: manic love, emotional dependence on the partner, and love addiction. The findings revealed unique correlational profiles for each construct and provided robust empirical support for our main hypothesis: these three behaviors, though related, are distinct psychological entities and must be investigated independently. Therefore, we strongly recommend that future research prioritize single-sample studies in which participants complete measures for all three constructs simultaneously. This approach is essential to definitively establish their discriminant and convergent validity using advanced statistical models and to resolve conceptual confusion.

Furthermore, moderation analysis provides critical insights into the role of gender/sex in these dynamics, demonstrating that the strength of certain correlations is significantly influenced by the gender/sex composition of the sample. Specifically, the associations between manic love and jealousy, manic love and relationship satisfaction, and love addiction and anxious attachment were all significantly moderated by the proportion of female participants.

The ultimate intention of this study is to move beyond the pathologization of problematic romantic behaviors. We align with authors who argue that problematic behaviors are not clinical pathologies or syndromes but rather stem from maladaptive cognitions, behaviors, and coping strategies that result in negative daily life consequences (Kardefelt‐Winther et al., 2017). Therefore, this systematic identification of specific correlates will allow advances in the study of their risk and protective factors.

These results provide a crucial theoretical and empirical basis for future professional interventions. Addressing and preventing these problematic romantic dynamics in a construct-specific and gender/sex-informed manner could help mitigate their associated negative consequences, ultimately supporting the creation of future prevention programs aimed at promoting healthier and safer relationships.

## Supplementary Information

Below is the link to the electronic supplementary material.

Supplementary file1 (DOCX 6482 kb)

## Declarations

### Generative AI Declaration

To ensure the highest linguistic quality, some paragraphs of this manuscript were refined with the assistance of Gemini and Paperpal. These tools were used exclusively to enhance clarity and fluency in English, while the scientific content and interpretations remain entirely our own.
