# Supplementary material for: Problematic Love Behaviors and Correlated Factors: A Systematic Review with Subgroup Meta-Analysis Including Gender/Sex Moderation
Source: Arch Sex Behav. 2026 Apr 24;55(3):1069–89. doi: 10.1007/s10508-026-03420-6 (PMC13194331; doi:10.1007/s10508-026-03420-6)

**Supplementary material**

**Table S1.**

*Descriptive characteristics of selected articles in alphabetical order (N =102)*

| **ID** | **Study** | **Country** | **Sample**  **(N, Mage, SD, %female)** | **Variable** | **Assessment tool** | **Correlates (association)** | **Quality^a^** |
| --- | --- | --- | --- | --- | --- | --- | --- |
| 1 | Aiquipa-Tello et al., 2024 | Peru | N = 1401 (75.8% female), M age = 21 (2.7) | ED | Emotional Dependence Inventory (IDE, Inventario de Dependencia Emocional, Aiquipa, 2012) | Intimate partner violence (+)  Life satisfaction (-) | Fair (3, 5, 14) |
| 2 | Arnold & Thompson, 1995 | US | N = 144 (80.6% female), M age = 21.24 (3.59) | ML | Mania subscale of the Love Attitudes Scale (LAS, Hendrick & Hendrick, 1986) | **MCMI-II scores** Dependent (NS) Debasement (+) Major Depression (+) Compulsive (NS) Alcohol Dependent (+) Disclosure (+) Borderline (+) Passive Aggressive (+) Desirability (NS) Drug Dependent (NS) Thought Disorder (NS) Self-defeating (+) Antisocial (NS) Avoidant (+) Dysthymic (+) Somatoform (+) Paranoid (NS) Anxious (+) Delusional (NS) Schizotypal (+) Schizoid (NS) Aggressive Sadistic (NS) Histrionic (NS) Narcissistic (NS) Bipolar/Manic (NS) | Fair (3, 5, 14) |
| 3 | Atlam et al., 2023 | Turkey | N = 1110 (61.4% female), M age = 21.2 (2.08) | LA | Addiction in Romantic Relationships Scale (Atlam et al., 2023) | Criteria for Addiction to love (+) Anxiety in relationships (+) Mother care (-) Mother protection (-) Father care (-) Father protection (-) | Good (14) |
| 4 | Barbarias et al., 2019 | Spain | N = 150 (76% female), M age = 27.81 (3.51) | ED | Emotional Dependence Questionnaire (CDE, Cuestionario de Dependencia Emocional, Lemos & Londoño, 2006) | Alcohol abuse (+)  Drug abuse (+)  **Attachment**  Security (NS)  Family concern (+)  Parental interference (+)  Parental authority value (+)  Parental permissiveness (NS)  Self-sufficiency and resentment towards parents (NS)  Childhood trauma (+) | Fair (3, 5, 14) |
| 5 | Barbarias et al., 2024 | Spain | N = 713 (81.5% female), M age = 21.27 (3.34) | ED | Emotional Dependency in Dating scale (DEN, Escala de dependencia emocional en el noviazgo de jóvenes y adolescentes, Urbiola et al., 2014) | **Social Media Addiction**  Obsession (+)  Lack of Personal Control in Social Media Use (+)  Excessive Social Media Use (+)  Overall Social Media Addiction (+)  **Attachment**  Security (NS)  Family Concern (NS)  Parental Interference (+)  Value of Parental Authority (+)  Parental Permissiveness (+)  Self-Sufficiency and Resentment Towards Parents (+)  Childhood Trauma (+) | Fair (3, 5, 14) |
| 6 | Blanchard & Fino, 2023 | UK | N = 599 (59.4% female). Age: > 18 years | ML | Mania subscale of the Love Attitudes Scale (LAS, Hendrick & Hendrick, 1986) | Primary psychopathy (+)  Secondary psychopathy (+)  Vulnerable narcissism (+)  Capacity for love (-) | Fair (3, 5, 14) |
| 7 | Bookwala et al. (1994) | US | N = 149 (100% female). Age: 94% 18 – 20 years old | ML | Mania subscale of the Love Attitudes Scale (LAS, Hendrick & Hendrick, 1986) | Aggression (+)  Satisfaction (-) | Fair (2, 3, 5, 14) |
| 8 | Borrello et al., 2023 | Italy | N = 409 (88% female), M age = 39.2 (14.92) | LA | Love Addiction Inventory (LAI, Costa et al., 2021) | **Perceived Social Support**  Family (NS) Friends (NS)  Significant Other (NS)  Total score (NS)  **Hypersex behavior** Coping (+)  Control (+)  Consequences (+) Total score (todo +) | Fair (2, 3, 5, 14) |
| 9 | Campbell et al., 2002 | US | Sample A: N = 80 (48.8% female), M age = 19.0; Sample B: N = 58 (53.4% female), M age = 19.0 | ML | Mania subscale of the Love Attitudes Scale (LAS, Hendrick & Hendrick, 1986) | Narcissism (NS) Self-esteem (-) | Good (5, 14) |
| 10 | Campos-Arregui et al., 2023 | Spain | N = 217 (70% female), M age = 21.77 (2.15) | ED | Affective Dependence Subscale of the Interpersonal Relationships and Emotional Dependencies Inventory (IRIDS-100, Inventario de Relaciones Interpersonales y Dependencias Sentimentales, Sirvent & Moral, 2018) | Alcohol consumption (NS) Depression (+) Anxiety (+)  General psychopathology (+) Internal Operating Models of the self (anxiety experienced in the face of abandonment or rejection) (-) Operational Models of others (avoidance of close relationships) (NS) | Fair (2, 5, 14) |
| 11 | Carone et al., 2025 | Italy | N = 505 (76.6% female), M age = 24.90 (2.67) | LA | Love Addiction Inventory (LAI, Costa et al., 2021) | **Male**  Emotional abuse (NS)  Physical abuse (NS)  Sexual abuse (NS)  Emotional neglect (NS)  Physical neglect (NS)  Vulnerable narcissism (+)  **Female**  Emotional abuse (+)  Physical abuse (NS)  Sexual abuse (NS)  Emotional neglect (+)  1211Physical neglect (+)  Vu12lnerable narcissism (+) | Good (5, 14) |
| 12 | Cassepp-Borges & Ferrer, 2019 | Brazil | N = 1549 (67.7% female), M age = 25.17 (7.74) | ML | Mania subscale of the Love Attitudes Scale (LAS, Hendrick & Hendrick, 1986) | Relat13ionship satisfaction (-) **Love14** Intimim15acy (-) Passion (+11) Comitment (NS) Love (NS) | Good (5, 14) |
| 13 | Castillo-Gonzáles & Terán Andrade, 2024 | Ecuador | N = 3202 (64.3% female), M age = 21.50 (2.82) | ED | Emotional Dependence Questionnaire (CDE, Cuestionario de Dependencia, Lemos & Londoño, 2006) | Victimization (+) Perpetration (+) | Fair (3, 5, 14) |
| 14 | Cavalli & Velotti, 2025 | Italy | Study 1: N = 720 (72.6% female), M age = 33.43 (13.07)  Study 2: N = 672 (71.6% female), M age = 33.43 (13.07) | LA | Love Addiction Inventory (LAI, Costa et al., 2021) | Study 1:  Anxiety adult attachment dimension (+)  Relational obsessive-compulsive disorder-total score (+)  Relational obsessive-compulsive disorder - love for partner (+)  Relational obsessive-compulsive disorder - adequacy of relationship (+)  Relational obsessive-compulsive disorder - partner’s love (+)  Study 2:  Difficulties in emotion regulation  in the dyadic context - lack of dyadic awareness (NS)  Difficulties in emotion regulation  in the dyadic context - lack of dyadic clarity (+)  Tendency  to enter tolerance-battering relationships – total score (+)  Tendency  to enter tolerance-battering relationships – insecure attachment (+)  Tendency  to enter tolerance-battering relationships – underserving self-image (+)  Tendency  to enter tolerance-battering relationships – self-sacrificing nature (+) | Good (3) |
| 15 | Chura et al., 2025 | Peru | N = 409 (49.9% female), M age = 35.95 (15.01) | ED | Emotional Dependence Questionnaire (CDE, Cuestionario de Dependencia, Lemos & Londoño, 2006) | Emotional Loneliness (NS)  Social Loneliness (NS)  Overall Feeling of Loneliness (NS) | Fair (3, 5, 14) |
| 16 | Coppolino et al., 2015 | Italy | N = 409 (49.9% female), M age = 35.95 (15.01) | ED | Emotional Dependence Questionnaire (CDE, Cuestionario de Dependencia, Lemos & Londoño, 2006) | Satisfaction of the romantic relationship (NS) Lenght (NS) | Fair (3, 5, 14) |
| 17 | Costa et al., 2021 | Italy | N = 329 (94.2% female), M age = 23.16 (2.77) | LA | Love Addiction Inventory (LAI, Costa et al., 2021) | Positive affect (-) Negative affect (+) | Fair (3, 5, 14) |
| 18 | Costa Galinha et al., 2013 | **Study 1:** North America (North Carolina, USA); **Study 2:** Africa (Maputo, Mozambique); **Study 3:** Europe (Lisbon, Portugal) | N = 1582; **Study 1:** N = 497 (64.3% female), M age = 19 (0.13); **Study 2:** N = 544 (42.8% female), M age = 25.18 (0.23); **Study 3:** N = 541 (56.4% female), M age = 23 (0.35) | ML | Love Attitudes Styles Short-Form (LAS-SF, Hendrick et al. 1998) | **Study 1** Attachment security (-) Satisfaction with current romantic relationships (NS) Satisfaction with romantic relationships (NS) Subjective well-being (-) **Study 2** Attachment security (-) Satisfaction with current romantic relationships (NS) Satisfaction with romantic relationships (NS) Subjective well-being (NS) **Study 3** Attachment security (-) Satisfaction with current romantic relationships (NS) Satisfaction with romantic relationships (-) Subjective well-being (-) | Fair (3, 5, 14) |
| 19 | Davies, 1995 | UK | N = 136 (65.4% female). M age = 30.67 (8.36) | ML | Mania subscale of the Love Attitudes Scale (LAS, Hendrick & Hendrick, 1986) | Self-esteem (-) Emotionality (+) Impulsivity (+) | Fair (3, 4, 5, 14) |
| 20 | de la Villa & López-Suárez, 2025 | Spain | N = 255 (female = 60.0%), M age = 29.73 (9.50) | ED | Affective Dependence Subscale of the Interpersonal Relationships and Emotional Dependencies Inventory (IRIDS-100, Inventario de Relaciones Interpersonales y Dependencias Sentimentales, Sirvent & Moral, 2018) | Negative feelings (+)  Loneliness (+)  Emptiness (+)  Self-destruction (+)  Inescapability (+)  Recreation of negative feelings (+)  Guilt (+) | Fair (3, 5, 14) |
| 21 | de la Villa & Prieto, 2022 | Spain | N = 627 (female = 74.7%), M age = 21.27 (1.72) | ED | Affective Dependence Subscale of the Interpersonal Relationships and Emotional Dependencies Inventory (IRIDS-100, Inventario de Relaciones Interpersonales y Dependencias Sentimentales, Sirvent & Moral, 2018) | Perpetrated cyber-control (+) Victimized cyber-control (+) Victimized cyber-agression (+) | Fair (2, 3, 5, 14) |
| 22 | de Oliveira Santos & Candeia Diniz, 2024 | Brazil | N = 200 (82.5% female), M age = 26.52 (8.95) | ED | Emotional Dependence Questionnaire (CDE, Cuestionario de Dependencia, Lemos & Londoño, 2006) | **Locus of control** Internality (+) Chance externality (-) Powerful Others externality (-) **Personality** Extroversion (-) Agreeableness (NS)  Conscientiousness (NS)  Neuroticism (+)  Openness (NS) | Fair (3, 5, 14) |
| 23 | Díaz et al., 2019 | Spain | N = 250 (100% female), M age = 58.66 (10.46) | ML | Mania subscale of the Love Attitudes Scale (LAS, Hendrick & Hendrick, 1986) | Overall negotiation received (NS)  Emotional negotiation received (NS)  Emotional negotiation perpetrated (+)  Cognitive negotiation received (NS)  Overall psychological aggression received (NS)  Overall psychological aggression perpetrated (NS)  Minor psychological aggression received (NS)  Minor psychological aggression perpetrated (+)  Severe psychological aggression received (NS)  Severe psychological aggression perpetrated (NS)  Overall physical assault received (NS)  Minor physical assault received (NS)  Minor physical assault perpetrated (NS)  Overall sexual coercion received (NS)  Overall sexual coercion perpetrated (NS)  Minor sexual coercion received (NS)  Minor sexual coercion perpetrated (NS)  Severe sexual coercion received (NS)  Severe injury received (NS) | Fair (3, 5, 14) |
| 24 | Dineen & Dinc, 2024 | UK | N = 249 (71 % female), M age = 39.51 (13.87) | LA | Love Addiction Inventory Short-Form (LAI-SF, Costa et al., 2021) | **Impulsive behavior** Positive Urgency (+) Negative Urgency (+) Lack of Perseverance (NS) Sensation Seeking (+) Lack of Premeditation (NS) **Difficulties in Emotion Regulation** Strategies (+) Non-Acceptance (+) Impulse (+) Goals (+) Awareness (NS) Clarity (+) |  |
| 25 | Dinić & Jovanović, 2021 | Serbia | N = 347 (73.5% females), M age = 24.42 (4.89) | ML | Mania subscale of the Love Attitudes Scale (LAS, Hendrick & Hendrick, 1986) | Narcissistic grandiosity (+) Narcissistic vulnerability (+) Narcissistic admiration (+) Narcissistic rivalry (+) Communal narcissism (+) | Good (14) |
| 26 | Eduardo Espinar & Rodríguez Hidalgo, 2015 | Spain | N = 100 (86% female), M age = 22.48 (2.82) | ED | Affective Dependence Subscale of the Interpersonal Relationships and Emotional Dependencies Inventory (IRIDS-100, Inventario de Relaciones Interpersonales y Dependencias Sentimentales, Sirvent & Moral, 2018) | **Social media use** Tuenti use (+) Youtube use (+) | Fair (3, 5, 14) |
| 27 | Ercan et al., 2025 | Turkey | N = 349 (76.5% female), M age = 23.59 (4.57) | ML | Love Attitudes Scale Short-Form (LAS-SF, Hendrick et al., 1998) | Intimacy (NS)  Relationship satisfaction (-) | Fair (3, 5, 14) |
| 28 | Erwin & Pressler, 2011 | UK | N = 143 (55.9% female), M age = 22.30 (3.30) | ML | Love Attitudes Scale Short-Form (LAS-SF, Hendrick et al., 1998) | Shyness (+) **Emotional Self-Disclosure Scale** Depression (NS) Happiness (NS) Jealousy (NS) Anxiety (NS) Anger (+) Calmness (NS)  Apathy (NS) Fear (NS) | Fair (3, 5, 14) |
| 29 | Estévez et al., 2017 | Spain | N = 535 (83.4% female), M age = 21.15 (2.48) | ED | Emotional Dependency in Dating scale (DEN, Escala de dependencia emocional en el noviazgo de jóvenes y adolescentes, Urbiola et al., 2014) | Anxiety (+) Depression (+) Self-esteem (-) Need for Exclusivity (+) Avoidance of Being Alone (+) Need to Please (+) Asymmetry (+) Mobile abuse (+) Internet abuse (+) | Fair (3, 5, 14) |
| 30 | Etxaburu et al., 2024a | Ecuador | N = 1498 (46.2% female), M age = 15.77 (1.21) | ED | Emotional Dependency in Dating scale (DEN, Escala de dependencia emocional en el noviazgo de jóvenes y adolescentes, Urbiola et al., 2014) | Impulsivity (+) | Good (14) |
| 31 | Etxaburu et al., 2024b | Spain | N = 711 (76.5% female), M age = 21.32 (2.94) | ED | Emotional Dependence Questionnaire (CDE, Cuestionario de Dependencia Emocional, Lemos & Londoño, 2006) | **Early dysfunctional schemas** Emotional deprivation (+) Abandonment (+) Mistrust (+) Failure (+) Dependence (+) Enmeshment (+) Subjugation (+) Emotional inhibition (+) Unrelenting standards (+) Entitlement (+) Insufficient self-control (+) Defectiveness (+) Self-sacrifice (+) Difficulty in emotion regulation (+) | Fair (3, 5, 14) |
| 32 | Flicker & Sancier-Barbosa, 2022 | US | N = 1831 (69.5% female), M age = 22.76 (6.52) | ML | The Love Attitudes Scale Short-Form (LAS-SF, Hendrick et al., 1998) | Attitudes toward polyamory (+) Willingness to engage in consensual non-monogamy (NS) | Good (5, 14) |
| 33 | Galicia Moyeda et al., 2013 | Mexico | N = 198 (54.0% female), aged 13 – 15 | ML | Mania subscale of the Love Attitudes Scale (LAS, Hendrick & Hendrick, 1986) | Violence received (+) Perpetrated violence (NS) | Fair (3, 5, 14) |
| 34 | García del Catillo-López et al., 2025 | Spain | N = 823 (87.6% female), M age = 32.54 (8.95) | ML | Mania subscale of the Love Attitudes Scale (LAS, Hendrick & Hendrick, 1986) | Emotional clarity (-)  Emotional repair (-)  Emotional flooding (+)  Conflict frequency (+)  Conflict intensity (+) | Good (3, 14) |
| 35 | Giacobbe et al., 2024 | Italy | N = 600 (74.3% female), aged 26 – 35 (50.83%) | LA | Love Addiction Inventory (LAI, Costa et al., 2021) | Age (-) Level of education (NS) Coping (+) Resilience (-) Perceived cognitive failures (+)  Perceived Memory Failures (+) Perceived Attentional Failures (+) Cognitive Function at Work (+) Anxiety (+) Depression (+) Frequency of memory failures (-) Social Media Addiction (+) | Good (5, 14) |
| 36 | Goodboy et al., 2010 | US | N = 205 (74.1% female), M age = 23.74 (9.07) | ML | Mania subscale of the Love Attitudes Scale Short Form (LAS-SF, Hendrick et al., 1998) | **Negative Relational Maintenance Behaviors** Jealousy Induction (+) Avoidance (+) Spying (+) Infidelity (+) Destructive Conflict (+) Allowing Control (+) **Relational Quality Indicator**s Satisfaction (-) Commitment (NS) Control Mutuality (-) Liking (NS) Respect (-) | Fair (3, 5, 14) |
| 37 | Goodboy et al., 2012 | US | N = 197 (52.8% female), M age = 19.81 (1.89) | ML | Mania subscale of the Love Attitudes Scale (LAS, Hendrick & Hendrick, 1986) | Jealousy evocation (NS) Affection received (NS) | Fair (3, 5, 14) |
| 38 | Gori et al., 2023 | Italy | N = 300 (80% female), M age = 37.83 (12.94) | LA | Love Addiction Inventory Short Form (LAI-SF, Costa et al., 2021) | Secure attachment (NS) Preoccupied attachment (+) Fearful attachment (+) Dismissing attachment (NS) Self-esteem (-) Age (-) Gender (NS) | Fair (3, 5, 14) |
| 39 | Gori et al., 2024a | Italy | N = 384 (66.15% female), M age = 25.90 (5.21) | LA | Love Addiction Inventory Short Form (LAI–SF, Costa et al., 2021) | Problematic Online Dating Apps Use (+) Social Media Addiction (+) Cyber Pornography Addiction (+) **Personality** Extraversion (NS) Agreeableness (-) Neuroticism (+) Openness (-) Conscientiousness (-) | Good (5, 14) |
| 40 | Gori et al., 2024b | Italy | N = 910 (76.6% female), M age = 30 (10.55) | LA | Love Addiction Inventory Short Form (LAI-SF, Costa et al., 2021) | **Family functioning**  Cohesion (-) Flexibility (NS) Disengagement (+) Enmeshment (+) Rigidity (+) Chaotic (+) **Childhood trauma** Emotional abuse (+) Physical abuse (+) Sexual abuse (NS) Emotional neglect (NS) Physical neglect (NS) | Fair (3, 5, 14) |
| 41 | Granda Cabal & Moral-Jimenez, 2022 | Spain | N = 263 (57.4% female), M age = 22.86 (2.71) | ED | Affective Dependence Subscale of the Interpersonal Relationships and Emotional Dependencies Inventory (IRIDS-100, Inventario de Relaciones Interpersonales y Dependencias Sentimentales, Sirvent & Moral, 2018) | Jealousy (+) Cyberviolence (+) Cyberaggression (+) Cybercontrol (+) | Fair (3, 5, 14) |
| 42 | Guthrie et al., 2018 | US | N = 436 (72.7% female). Age: A majority of the sample was 19 years or younger (80%),followed by ages 20 to 22 (16%). | ML | Mania subscale of the Love Attitudes Scale Short Form (LAS-SF, Hendrick et al., 1998) | Possitive pet attitudes (NS) Negative pet attitudes (+) Self-esteem (NS) Relationship satisfaction (-) Social support (NS) | Fair (3, 5, 14) |
| 43 | Hetsroni, 2012 | Israel | N = 338 (67.5% female), M age = 23.3 (2.6) | ML | Mania subscale of the Love Attitudes Scale (LAS, Hendrick & Hendrick, 1986) | TV Viewing (NS) | Good (5, 14) |
| 44 | Huerta-Rosales et al., 2025 | Peru | N = 555 (50.1% female), Age:  94.8% 18-29 years old,  5.2% 30-59 years old | ED | Emotional Dependence Questionnaire (CDE, Cuestionario de Dependencia Emocional, Lemos & Londoño, 2006) | Intimate partner violence (+) | Fair (3, 5, 14) |
| 45 | Jiménez-Cruz et al., 2022 | Mexico | N = 559 (50.1% female), M age = 20.53 (1.88) | ED | Emotional Dependence Scale (Jiménez-Cruz et al., 2022) | Violence (+) Social support perceived (-) | Good (3, 14) |
| 46 | Jonason & Kavanagh, 2010 | 15% Australian, 3% Canadian, 9% New Zealander, 70% American, and less than 1% German, British, Italian, Dutch, Puerto Rican, Turkish, and from the United Arab Emirates. | N = 302 (81.1% female), M age = 28.54 (10.73) for males; M age = 27.23 (9.68) for females. | ML | Mania subscale of the Love Attitudes Scale (LAS, Hendrick & Hendrick, 1986) | **Dark triad** Machiavellianism (+) Narcissism (NS) Psychopathy (NS) Dark Triad composite (NS) | Good (5, 14) |
| 47 | Jonason et al., 2020 | US | N = 311 (88.1% female) M = 16.01; SD = 1.15 | ML | Mania subscale of the Love Attitudes Scale (LAS, Hendrick & Hendrick, 1986) | **Pathological personality traits** Antagonism (+) Psychoticism (+) Detachment (+) Negative Affectivity (+) Disinhibition (+) | Fair (3, 5, 14) |
| 48 | Jones & Nelson, 1997 | US | N = 307 (71.7% female). Age: 58% were between 17 and 19 years old; 33.3% were 20 to 21 years of age; and 8.8% were 22 years old and above. | ML | Mania subscale of the Love Attitudes Scale (LAS, Hendrick & Hendrick, 1986) | Romanticism (+) | Fair (3, 5, 14) |
| 49 | Kanemasa et al., 2004 | Japan | N = 343 (46.3% female), M age = 19.00 (0.85) | ML | Mania subscale of the Love Attitudes Scale (LAS, Hendrick & Hendrick, 1986) | **Emotional experiences**  **Male**  Hostility (NS)  Affection (+)  Relaxation (NS)  Uncertainty (NS)  Anxiety/Jealousy (+)  Liveliness (+)  Politeness (NS)  Envy (+)  **Female**  Hostility (NS)  Affection (+)  Relaxation (NS)  Uncertainty (+)  Anxiety/Jealousy (+)  Liveliness (+)  Politeness (NS)  Envy (+) | Good (3, 14) |
| 50 | Kemer et al., 2016 | Turkey | N = 384 (52.9% female), M age = 35.98 (8.00) | ED | Emotional Dependency Scale (EDS, Buunk, 1981) | Relationship satisfaction (+) Interpersonal Rejection (NS) Interpersonal Misperception (+) Unrealistic Relationship Expectations (+) Gender (NS) Length marriage (NS) | Fair (3, 4, 5, 14) |
| 51 | Kunkel & Burleson, 2003 | US | N = 272 (56.3% female), Age: 63.2% of the sample was between the ages of 19 and 21, 32% of the sample was 22 and over, and 4.8% of the sample was 18 years old. | ML | Mania subscale of the Love Attitudes Scale (LAS, Hendrick & Hendrick, 1986) | Sex (NS) **Communication values Affectively oriented skills** Conflict management (+) Comforting (+)  Ego support (+)  Regulative skill (+)  **Instrumentally oriented skills** Persuasive (+) Conversational (+) Referential skill (+) Narrative skill (NS) | Fair (3, 5, 14) |
| 52 | Láng et al., 2021 | Hungry | N = 481 (34.3% female), M age = 47.8 (6.46). | ML | Love Attitudes Scale – Short Form (LAS-SF, Hendrick et al., 1998) | ASR-OMWS, Acceptance of Sugar Relationship in Older Men and Women Scale (NS) | Fair (3, 5, 14) |
| 53 | Levine et al., 2007 | US | **Study 1**: N = 108 (64.8% female); **Study 2:** N = 173 (43.4% female); **Study 3:** N = 137 (71.7% female) | ML | Mania subscale of the Love Attitudes Scale (LAS, Hendrick & Hendrick, 1986) | **Study 1:** **Attractive Partner Characteristics** Honesty (NS) Looks (NS) Humor (NS) Smart (-) Understand (NS) Personality (NS) Compassion (NS) Communicate (NS) Caring (NS) Sensitive (+) Money (NS) Sex (-) Success (NS) Romantic (NS)  **Study 2:** **Opening Line Types** Direct (NS) Innocuous (+) Cute (NS) I**ntensification Strategy Type** Increased Contact (NS) Relational Negotiation (NS) Social Support and Assistance (+) Increased Rewards (+) Direct Definitional Bid (NS) Tokens of Affection (+) Personalized Communication (+) Verbal Expressions of Affection (NS) Suggestive Actions (NS) Nonverbal Expressions of Affection (+) Social Enmeshment (NS) Personal Appearance (+) Sexual Intimacy (NS) Behavioral Adaptation (+)  **Study 3:** **Secret Tests** Third Party (NS) Triangle (NS) Direct (-) Separation (+) Endurance (+) Public (NS) Indirect (+) | Fair (3, 5, 14) |
| 54 | Macía et al., 2022 | Ecuador | N = 1533 (46.1% female), M age = 15.76 (1.25) | ED | Emotional Dependency in Dating scale (DEN, Escala de dependencia emocional en el noviazgo de jóvenes y adolescentes, Urbiola et al., 2014) | Physical violence received (+) Sexual violence received (+) Psychological-social violence received (+) Psychological-humiliation violence received (+) Psychological-jealousy violence received (+) Received violence (+) Alcohol addiction (+) Gambling addiction (+) Drugs addiction (+) Eating disorder (+) Internet addiction (+) Gaming addiction (+) Compulsive spending (+) Sex addiction (+) | Good (3, 14) |
| 55 | Marchi et al., 2023 | Italy | N = 274 (82.0% female), M age = 27.89 (8.39). | ML | Mania subscale of the Love Attitudes Scale Short Form (LAS-SF, Hendrick et al., 1998) | **24 ways to be compatible** Lifestyle (NS) Opinions (NS) Emotions (NS) Origins (NS) Sociality (NS) Romanticism (+) Morals (NS) Family (NS) Food (NS) Sensation (-) Class (NS) Religion (NS) Conformity (-) Leisure (NS) Appearance (NS) Job (NS) Conflict (NS) Empathy (NS) Humor (NS) Residence (NS) Speech (NS) Intellect (NS) Enthusiasm (NS) Activity (NS) | Good (14) |
| 56 | Marzec &  Łukasik, 2017 | Poland | N = 177 (50.8% female), M age = 27.89 (7.04) | ML | Mania subscale of the Love Attitudes Scale Short Form (LAS-SF, Hendrick et al., 1998) | Family Environment Stability Index (NS) Type of reproductive strategy (NS) Socio sexual behavior (NS) Socio sexual attitude (NS) Socio sexual desire (+) Socio sexual global score (NS) | Good (5, 14) |
| 57 | Mattingly et al., 2012 | US | N = 199 (73.4% female), M age = 19.40 (2.40) | ML | Love Attitudes Styles Short Form (LAS-SF; Hendrick et al. 1998) | Romantic Jealousy-Induction (+) Testing/Strengthening Relationship (+) Revenge - Motives for Inducing Romantic Jealousy (+) Power/Control - Motives for Inducing Romantic Jealousy (+) Security -Motives for Inducing Romantic Jealousy (+) Self-Esteem - Motives for Inducing Romantic Jealousy (+) Thoughts - Multidimensional Jealousy (+) Emotions - Multidimensional Jealousy (+) Behaviors - Multidimensional Jealousy (+) Attachment Avoidance - Experiences in Close Relationships (NS) Attachment Anxiety - Experiences in Close Relationships (+) Satisfaction - Investment Model (NS) Alternatives - Investment Model (NS) Investments - Investment Model (+) Commitment - Investment Model (NS) | Fair (3, 5, 14) |
| 58 | McCutcheon, 2002 | US | N = 107 (33.6% female), M age = 20.52 (4.12) for males; M age = 21.78 (5.63) for females. | ML | Love Attitudes Scale Short Form (LAS-SF, Hendrick et al., 1998) | The CAS measures the favorability of attitudes toward one's favorite celebrity. Social/Entertainment (NS) Intense/Personal (+) Mild Pathology (NS) Total CAS (+) | Fair (3, 5, 14) |
| 59 | McCutcheon et al., 2016 | US | N = 330 (74.0% female), M age = 20.97 (4.82) | ML | Mania subscale of the Love Attitudes Scale Short Form (LAS-SF, Hendrick et al., 1998) | **Celebrity Attitude Scale** Entertainment-Social (+) Intense-Personal (+) Borderline Pathological (+) **Romantic Partner Conflict Scale** Compromise (NS) Domination (+) Submission (+) Separation (NS) Avoidance (NS) Interactional Reactivity (+)  Experiences in Close Relationship Scale-Anxiety (+) Relationship Theories Questionnaire - Soulmate (+) | Fair (3, 5, 14) |
| 60 | Meskó et al., 2021 | Hungry | N = 800 (54.9% female), M age = 38.6 (12.5) | ML | Mania subscale of the Love Attitudes Scale Short Form (LAS-SF, Hendrick et al., 1998) | Sociosexual Orientation Behavior (NS) Attitude (-) Desire (NS) Total (NS) Mate Value (NS) Reasons for having sex Personal Goal Attainment (+) Relational Reasons (+) Sex as Coping (+) | Fair (3, 5, 14) |
| 61 | Michalska et al., 2023 | US | N = 308 (56.3% female), M age = 33.53 (11.15) | ML | Mania subscale of the Love Attitudes Scale (LAS, Hendrick & Hendrick, 1986) | **Personality Five Factor model of the DSM** Disinhibition (+) Detachment (+) Psychoticism (+) Negative Affect (+) Antagonism (+)  Loneliness (+) Self-Esteem (-) | Good (3, 14) |
| 62 | Miller et al., 2010 | US | N = 238 (60.0% female), M age = 19.13 (1.26) | ML | Mania subscale of the Love Attitudes Scale (LAS, Hendrick & Hendrick, 1986) | Elemental Psychopathy total (NS) Unconcern (-) Anger/Hostility (+) Self-contentment (-) Self-assurance (NS) Urgency (+) Invulnerability (-) Coldness (NS) Dominance (NS) Thrill Seeking (NS) Distrust (NS) Manipulation (NS) Self-centeredness (NS) Opposition (NS) Arrogance (NS) Callousness (NS) Disobliged (NS) Impersistence (+) Rashness (NS) | Good (14) |
| 63 | Miller et al., 2012 | US | N = 287 (60.0% female), M age = 18.9 (1.16). | ML | Mania subscale of the Love Attitudes Scale (LAS, Hendrick & Hendrick, 1986) | **Five-Factor Narcissism Inventory (FFNI)** Reactive anger (+) Shame (NS) Indifference (NS) Need for admiration (NS) Exhibitionism (NS) Authoritativeness (NS) Thrill seeking (+) Grandiose fantasies (NS) Cynicism/distrust (+) Manipulativeness (+) Exploitativeness (NS) Entitlement (+) Lack of empathy (NS) Arrogance (+) Acclaim seeking (NS) FFNI Grandiose (+) FFNI Vulnerable (+) | Fair (3, 5, 14) |
| 64 | Momeñe et al., 2021 | Spain | N = 1533 (46.1% female), M age = 15.76 (1.25) | ED | Emotional Dependency in Dating scale (DEN, Escala de dependencia emocional en el noviazgo de jóvenes y adolescentes, Urbiola et al., 2014) | **Consumption last 30 days: frequency** Tobacco (+) Alcohol (+) Wine/Champagne or cava (working days) (NS) Beer/Cider (working days) (NS) Appetizers/vermouth (working days) (NS) Combinations/cubates (working days) (NS) Fruit liqueurs (working days) (+) Hard liquor (working days) (NS) Wine/champagne or cava (weekends) (NS) Beer/Cider (weekends) (+) Fruit liqueurs (weekends) (+) Hard liquor (weekends) (NS) Tranquilizers/sedatives or sleeping pills (+) Over-the-counter tranquilizers/sedatives or sleeping pills (+) Hashish or marijuana (+) Cocaine in base (+) Cocaine powder (+) GHB or liquid ecstasy (+) Ecstasy (+) Amphetamines or speed (+) Hallucinogens (+) Heroin (+) Volatile inhalants (+) **Consumption last 30 days: quantity** Beer/Cider (working days) (+) Hard liquor (working days) (NS) Wine/champagne or cava (weekends) (NS) Beer/Cider (weekends) (+) Fruit liqueurs (weekends) (+) Hard liquor (weekends) (+) 5 or more glasses of alcohol on the same occasion (+) Get drunk (+) | Good (3, 14) |
| 65 | Momeñe & Estévez, 2018 | Spain | N = 269 (81.6% female), M age = 28.34 (10.15) | ED | Affective Dependence Subscale of the Interpersonal Relationships and Emotional Dependencies Inventory (IRIDS-100, Inventario de Relaciones Interpersonales y Dependencias Sentimentales, Sirvent & Moral, 2018) | Psychological Abuse (+) Security, Availability, Support (-) Secure Attachment (-) Preoccupation with Family (+) Parental Interference (+) Anxious Attachment (+) Value of Authority (NS) Permissiveness (+) Self-Sufficiency and Resentment (+) Avoidant Attachment (+) Childhood Trauma (+) Disorganized Attachment (+) Dependency and Psychological Abuse | Fair (3, 5, 14) |
| 66 | Momeñe & Estévez, 2019 | Spain | N = 299 (79.3% female), M age = 29.53 (10.81) | ED | Affective Dependence Subscale of the Interpersonal Relationships and Emotional Dependencies Inventory (IRIDS-100, Inventario de Relaciones Interpersonales y Dependencias Sentimentales, Sirvent & Moral, 2018) | Psychological abuse (+) Resilience (-) | Fair (2, 3, 5, 14) |
| 67 | Momeñe López et al., 2022a | Spain | N = 258 (77.1% female), M age = 32.63 (11.66) | ED | Emotional Dependence Questionnaire (CDE, Cuestionario de Dependencia Emocional, Lemos & Londoño, 2006) | Social anxiety (+) Fear of negative evaluation (+) Dysfunctional perfectionism (+) Physical violence (+)  Psychological violence (+) | Fair (3, 5, 14) |
| 68 | Momeñe López et al., 2022b | Spain | N = 258 (77.1% female), M age = 32.63 (11.66) | ED | Emotional Dependence Questionnaire (CDE, Cuestionario de Dependencia Emocional, Lemos & Londoño, 2006) | Physical Violence Received (+) Psychological Violence Received (+) Intolerance of Uncertainty (+) Inhibition-Generating Uncertainty (+) Uncertainty as Bewilderment and Unpredictability (+) Tendency to Worry (+) Pessimism (+) | Fair (3, 5, 14) |
| 69 | Nazzal et al., 2021 | Palestine | N = 500 (63.6% female), Age: 21 – 26 | ML | Mania subscale of the Love Attitudes Scale (LAS, Hendrick & Hendrick, 1986) | **Male**  Loneliness (NS)  Satisfaction with Love Life (NS)  **Female**  Loneliness (NS)  Satisfaction with Love Life (NS) | Fair (3, 5, 14) |
| 70 | Neto & Pinto, 2003 | Portugal | N = 315 (62.5% female), M age = 17.10 (0.98) | ML | Mania subscale of the Love Attitudes Scale Short Form (LAS-SF, Hendrick et al., 1998) | Loneliness (NS) | Fair (3, 5, 14) |
| 71 | Neto & Pinto, 2025 | Portugal | N = 1153 (48.0% female), M age = 38.00 (17.3) | ML | Mania subscale of the Love Attitudes Scale Short Form (LAS-SF, Hendrick et al., 1998) | Satisfaction with love life (NS)  Satisfaction with sex life (NS)  Sexual desire (+)  Commitment (+)  Romantic loneliness (NS) | Fair (3, 5, 14) |
| 72 | Neto, 1992 | Portugal | N = 185 (50.8% female), M age = 22.10 (2.70) | ML | Mania subscale of the Love Attitudes Scale (LAS, Hendrick & Hendrick, 1986) | Private self-consciousness (+) Public self-consciousness (+) Social anxiety (+) Public performing (-) Others directedness (NS) | Fair (2, 3, 5, 14) |
| 73 | Neto, 2015 | Portugal | N = 475 (42.0% female), M age = 37.28 (16.27) | ML | Mania subscale of the Love Attitudes Scale (LAS, Hendrick & Hendrick, 1986) | Sociosexuality (-) | Fair (3, 5, 14) |
| 74 | Odilavadze et al., 2019 | US | N = 87 (49.4% female), M age = 32.80 (11.40). | ML | Mania subscale of the Love Attitudes Scale (LAS, Hendrick & Hendrick, 1986) | Marital satisfaction (NS) | Fair (2, 3, 5, 14) |
| 75 | Olave et al., 2021 | Spain | N = 366 (54.6% female), M age = 23.53 (6.48) | ED | Emotional Dependence Questionnaire (CDE, Cuestionario de Dependencia Emocional, Lemos & Londoño, 2006) | Separation Anxiety (+) Partner's Affective Expression (+) Changing of Plans (+) Fear of Loneliness (+) Borderline Expression (+) Attention Seeking (+) Exercise Addiction (+) Muscle Dysmorphia (+) Muscle Satisfaction (-) Substance Use (+) Muscle Checking (+) Injury (+) Exercise Dependence (+) Security (NS) Preoccupation with Family (NS) Parental Interference (+) Regard of Authority (+) Parental Permissiveness (NS) Self-Sufficiency and Resentment Toward Parents (+) Childhood Trauma (+) | Fair (3, 5, 14) |
| 76 | Olave et al., 2024 | Spain | N = 887 (86% female), M age = 20.82 (3.63) | ED | Emotional Dependency in Dating scale (DEN, Escala de dependencia emocional en el noviazgo de jóvenes y adolescentes, Urbiola et al., 2014) | Exercise Addiction (+) Self-Esteem (-) Impulsivity (+) Received Violence (+) Exerted Violence (+) Perceived Violence (-) | Fair (3, 5, 14) |
| 77 | Petruccelli et al., 2014 | Italy | N = 3375 (50.3% female), M age = 28.64 (8.99) | ED | Emotional Dependency subscale of the Spouse-Specific Dependency Scale (Rathus & O'Leary, 1997) | Proactive agression (+) | Fair (3, 5, 14) |
| 78 | Proyer et al., 2018 | Switzerland | N = 240 (61,2% female), M age = 26.8, (9.2) | ML | Marburg Attitude Inventory for Love Styles (MAIL; Bierhoff et al., 1993) | **Playfulness**  **Male**  Short Measure of Adult Playfulness (NS)  Other-directed (NS)  Lighthearted (NS)  Intellectual (NS)  Whimsical Playfulness (NS)  **Female**  Short Measure of Adult Playfulness (NS)  Other-directed (NS)  Lighthearted (NS)  Intellectual (NS)  Whimsical Playfulness (NS) | Fair (3, 5, 14) |
| 79 | Robles Ojeda et al., 2021 | Mexico | N = 291 (53.9% female). Secondary level: M age = 14.6. Baccalaureate: Mage = 16.9 años. Undergraduates: M age = 19.0 | ML | Mania subscale of the Love Attitudes Scale (LAS, Hendrick & Hendrick, 1986) | Economic Abuse (+)  Psychological Abuse (+)  Sexual Abuse (+)  Physical Abuse (+) | Fair (3, 5, 14) |
| 80 | Rodriguez Perez et al., 2019 | Spain | N = 776 (75.4% female), M age = 22.30 (6.21) | ED | Emotional Dependence Questionnaire (CDE, Cuestionario de Dependencia Emocional, Lemos & Londoño, 2006) | **Conflict resolution styles Exercised** Negotiation (NS) Psychological abuse (+) Physical aggression (+) Sexual coercion (+) Injury (-) **Received** Negotiation (NS) Psychological abuse (+) Physical aggression (NS) Sexual coercion (+) Injury (NS) | Fair (3, 5, 14) |
| 81 | Rohmann et al., 2012 | Germany | N = 92 (66.3% female), M age = 24.00 (5.00). | ML | Marburg Attitude Inventory for Love Styles (MAIL; Bierhoff et al., 1993) | Grandiose narcissism (NS) Vulnerable narcissism (+) | Fair (3, 4, 5, 14) |
| 82 | Sanrı & Goodwin, 2013 | Turkey and UK | N = 224 (57.1% female), Age: Turkish sample: M age = 32.50 (11.45), British sample: M age = 25.60 (8.36) | ML | Mania subscale of the Love Attitudes Scale Short Form (LAS-SF, Hendrick et al., 1998) | **Individual-level values** Conservation (-) Openness to Change (-) Self-Transcendence (NS) Self-Enhancement (NS) Hedonism (NS) | Fair (3, 5, 14) |
| 83 | Sarwer et al., 1993 | US | N = 63 (0% female) | ML | Mania subscale of the Love Attitudes Scale (LAS, Hendrick & Hendrick, 1986) | Sexual experience (NS)  Sociopathy (NS)  Masculinity (NS)  Likely rape (NS)  Likely force (NS) | Fair (3, 5, 14) |
| 84 | Schreurs & Buunk, 1996 | Netherlands | N = 234 (100% female), M age = 34.0 (7.10) | ED | Emotional Dependency Scale (EDS, Buunk, 1981) | Total intimacy (+)  Intimate disclosure (+)  Social intimacy (+)  Recreational intimacy (+)  Sexual intimacy (+)  Autonomy (-)  Equity (NS) | Good (5, 14) |
| 85 | Shurts & Myers, 2008 | US | N = 168 (75.0% female), M age = 25.12 (7.76). | ML | Love Attitudes Scale Short Form (LAS-SF, Hendrick et al.,1998) | Liking (platonic, interpersonal relationshipthat does not involve a marital, sexual, or familial commitment) (NS) **Wellness** Creative self (NS) Coping self (-) Social (NS) Friendship (NS) Love (NS) Essential (-) Physical (NS) Total Wellness (-) | Fair (3, 5, 14) |
| 86 | Smith & Klases, 2016 | UK, Hong Kong | N = 108 (57% female), M age = 28.96 (ranging from 20-61) | ML | Mania subscale of the Love Attitudes Scale Short Form (LAS-SF, Hendrick et al., 1998) | Gender (NS) Length (NS) Collectivism (NS) Individualism (NS) **Attachment** Anxious (-) Avoidant (-) | Fair (3, 5, 14) |
| 87 | Soares et al., 2020 | Portugal | N = 357 (57.0% female). Two age groups were considered: 18–21 years (65%) and 22–27 years (35%). | ML | Mania subscale of the Love Attitudes Scale (LAS, Hendrick & Hendrick, 1986) | Satisfaction with love life (-) | Fair (3, 5, 14) |
| 88 | Stead et al., 2021 | UK | N = 151 (100% female), M age = 23.34 (8.80) | ML | Love Attitudes Scale Short Form (LAS-SF, Hendrick et al.,1998) | Borderline Personality (-)  Histrionic Personality (-)  Rejection Sensitivity (-)  Nonverbal Sexual  Arousal Perpetration (-)  Emotional Manipulation and Deception Perpetration (-)  Exploitation of the Intoxicated Perpetration (NS)  Nonverbal Sexual Arousal Victimisation (-)  Emotional Manipulation and Deception Victimisation (NS)  Exploitation of the Intoxicated Victimisation (NS) | Good (3, 14) |
| 89 | Telli & Yavuz Güler, 2023 | Turkey | N = 240 (50% female), M age = 33.56 (7.86) for females; 36.57 (8.93) for males | ED | Emotional Dependency Scale (EDS, Buunk, 1981) | Differentiation of self (NS) Relationship satisfaction (+) Relationship adjustment (+) Forgiveness (+) Jealousy (NS) **Conflict resolution responses (+)** Exit response (-) Neglect response (-) | Fair (3, 5, 14) |
| 90 | Topino et al., 2023 | Italy | N = 332 (80% female), M age = 23.00 (2.46) | LA | Love Addiction Inventory Short Form (LAI-SF, Costa et al., 2021) | Secure Attachment (NS)  Preoccupied Attachment (NS)  Fearful Attachment (+)  Dismissing Attachment (NS)  Separation Anxiety (+)  Mature Defenses (NS)  Neurotic Defenses (+)  Immature Defenses (+) | Fair (3, 5, 14) |
| 91 | Tošić-Radev & Hedrih, 2017 | Serbia | N = 500 (79.0% female). Aged 18 – 40 (94.8 % of the sample belonged to the age group from 25 – 35 years) | ML | Love Attitudes Scale (LAS, Love Attitudes Scale, Hendrick & Hendrick, 1986) | Cognitive jealousy (+) Emotional jealousy (+) Behavioral jealousy (+) | Fair (3, 5, 14) |
| 92 | Urbiola et al., 2014 | Spain | N = 761 (82.2% female), M age = 16.80 (3.40) | ED | Emotional Dependency in Dating scale (DEN, Escala de dependencia emocional en el noviazgo de jóvenes y adolescentes, Urbiola et al., 2014) | **Dysfunctional schemas**  Dependency (+)  Subjugation (+)  Attachment (+)  Abandonment (+)  Self-sacrifice (+)  Emotional deprivation (+) | Fair (3, 5, 14) |
| 93 | Urbiola et al., 2019 | Spain | N = 550 (84% female), M age = 21.16 (2.41) | ED | Emotional Dependency in Dating scale (DEN, Escala de dependencia emocional en el noviazgo de jóvenes y adolescentes, Urbiola et al., 2014) | Self-esteem (-)  Psychological-Social Violence Received (+)  Psychological Violence - Humiliation Received (+)  Psychological Violence-Control Received (+)  Psychological-Social Violence Executed (+)  Psychological Violence - Humiliation Exercised (+)  Psychological Violence-Control Exercised (+) | Fair (3, 5, 14) |
| 94 | Vedes et al., 2016 | Switzerland | N = 184 (50% females), M age = 36.1 (10.5) | ML | Marburg Attitude Inventory for Love Styles (MAIL; Bierhoff et al., 1993) | **Male**  Supportive dyadic coping (NS)  Common dyadic coping (NS)  Relationship satisfaction (NS)  Relationship length (NS)  **Female**  Supportive dyadic coping (NS)  Common dyadic coping (NS)  Relationship satisfaction (NS)  Relationship length (NS) | Fair (3, 5, 14) |
| 95 | Villora et al., 2019 | Spain | N = 1657 (62.9% female), M age = 20.59 (3.21) | ED | Emotional Dependency subscale of the Spouse-Specific Dependency Scale (Rathus & O'Leary, 1997) | Direct victimization (+) Control victimization (+) Direct perpetration (+) Control perpetration (+) Exclusive dependency (+) Anxious attachment (+) Social support (+) Social skills (+) Planning behavior (+) Goal efficacy (+) | Fair (3, 5, 14) |
| 96 | Wang et al., 2025 | China | N = 464 (64.9% female), M age = 22.29 (2.13) | LA | Love Addiction Inventory (LAI, Costa et al., 2021) | Sense of giving (+)  Relationship power (-)  Perceived acceptability of gaslighting (+) | Fair (3, 5, 14) |
| 97 | Wan Shahrazad et al., 2012 | Malaysia | N = 200 (50.0% female), Age = 20 – 25 | ML | Love Attitudes Scale Short Form (LAS-SF. Hendrick et al., 1998) | **Sexual attitudes** Permissiveness (NS) Birth control (NS) Communion (+) Instrumentality (NS) | Fair (3, 5, 14) |
| 98 | White et al., 2004 | US | N = 196 (53.6% female), Age: 63.8% were 18–19, 32.1% were 20–22, and 4.1% were 23–30 | ML | Mania subscale of the Mania subscale of the Love Attitudes Scale Short Form (LAS-SF, Hendrick et al., 1998) | **Big five** Neuroticism (+) Extraversion (NS) Openness (NS) Agreeableness (NS) Conscientiousness (NS) | Fair (3, 5, 14) |
| 99 | Worobey, 2001 | US | N = 244 (66.8% female), M age = 20.49 (3.38) | ML | Mania subscale of the Mania subscale of the Love Attitudes Scale (LAS, Hendrick & Hendrick, 1986) | **Temperament** Activity (NS) Distress (+) Fearfulness (+) Sociability (NS) Anger (NS) | Fair (3, 5, 14) |
| 100 | Yıldız-Önal & Uçar, 2023 | Turkey | N = 400 (59.5% female), M age = 35.50 (7.68) | ED | Emotional Dependency Scale (EDS, Buunk, 1981) | Communication skills (NS) Partner accommodation (NS)  Relationship belief (+) | Fair (3, 5, 14) |
| 101 | Zeigler-Hill et al., 2014 | US | N = 385 (70.5% female), M age = 21.3 (3.52) | ML | Mania subscale of the Love Attitudes Scale (LAS, Hendrick & Hendrick, 1986) | Self-Esteem Level (-) Self-Esteem Instability (+) | Good (5, 14) |
| 102 | Zibenberg & Natividade, 2025 | Brazil | N = 1310, M age = 30.25 years (12.2)  Self-esteem: N = 639 | LA | Love Addiction Inventory (LAI, Costa et al., 2021) | Age (-)  Length (-)  Meeting frequency (-)  Self-esteem (-) | Fair (3, 5, 14) |
|  | ^a^ Overall quality of studies based on the quality criteria from Table 2 (NHLBI et al., 2014). Numbers in brackets represent unmet criteria.  ED: Emotional dependence, ML: Manic love, LA: Love addiction.  + : Positive association, - : Negative association, NS: Non-significative association. | | | | | | |

**Fig. 1S.**

*Forest plot for the association between manic love and self-esteem.*


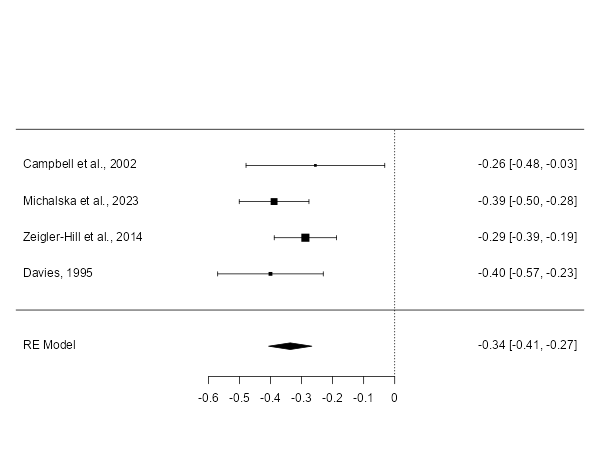


**Fig. 2S.**

*Forest plot for the association between manic love and narcissism.*


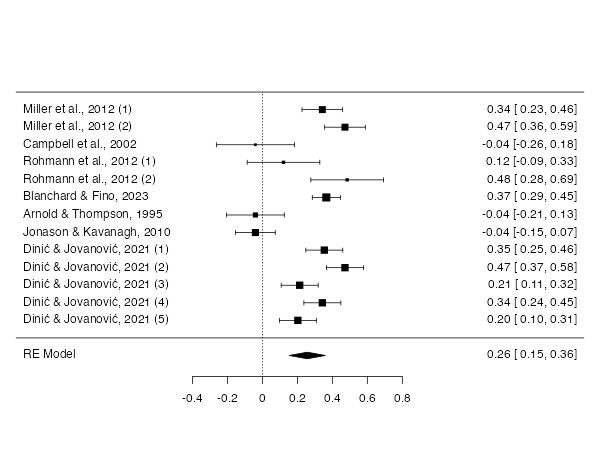


**Fig. 3S.**

*Forest plot for the association between manic love and psychopaty.*


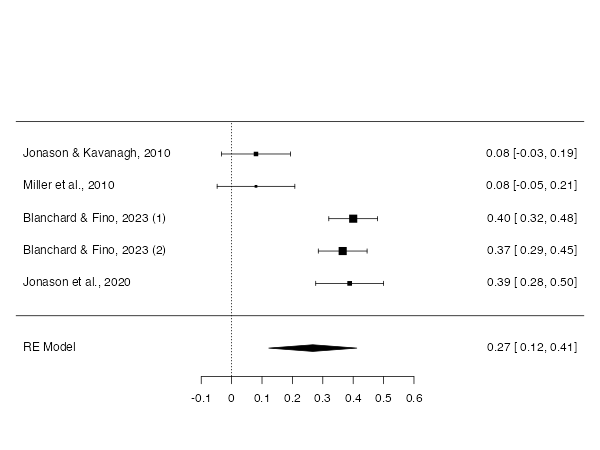


**Fig. 4S.**

*Forest plot for the association between manic love and jealousy.*


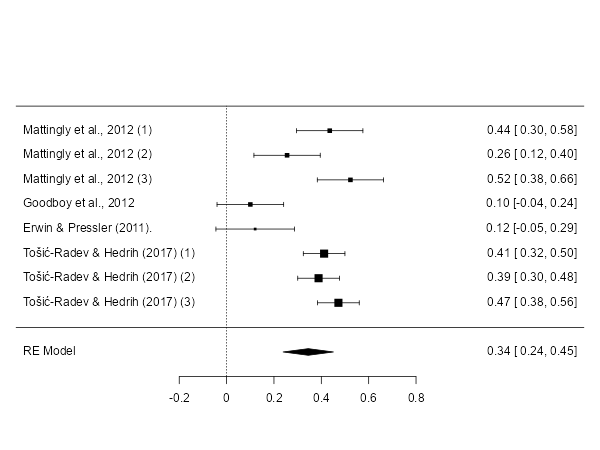


**Fig. 5S.**

*Forest plot for the association between manic love and violence perpetrated.*


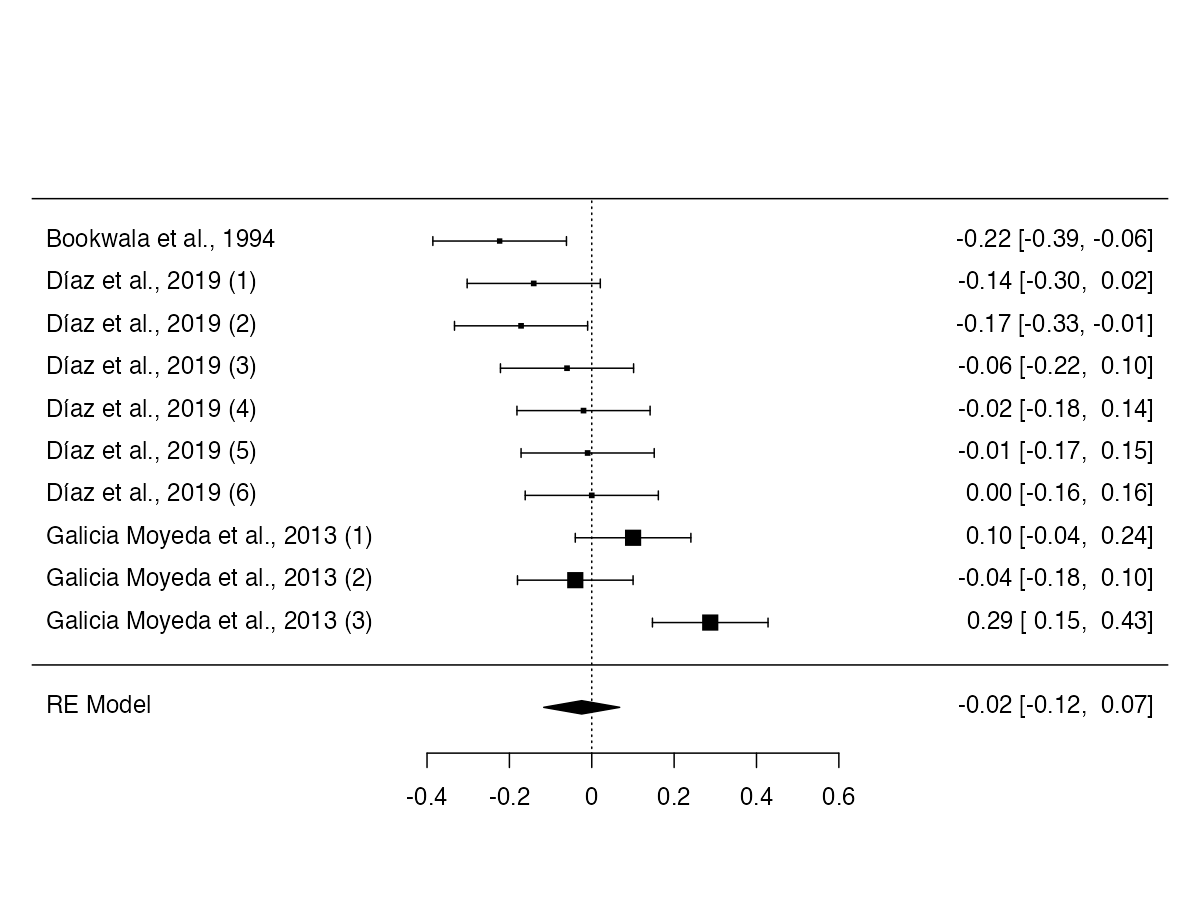


**Fig. 6S.**

*Forest plot for the association between manic love and relationship satisfaction.*


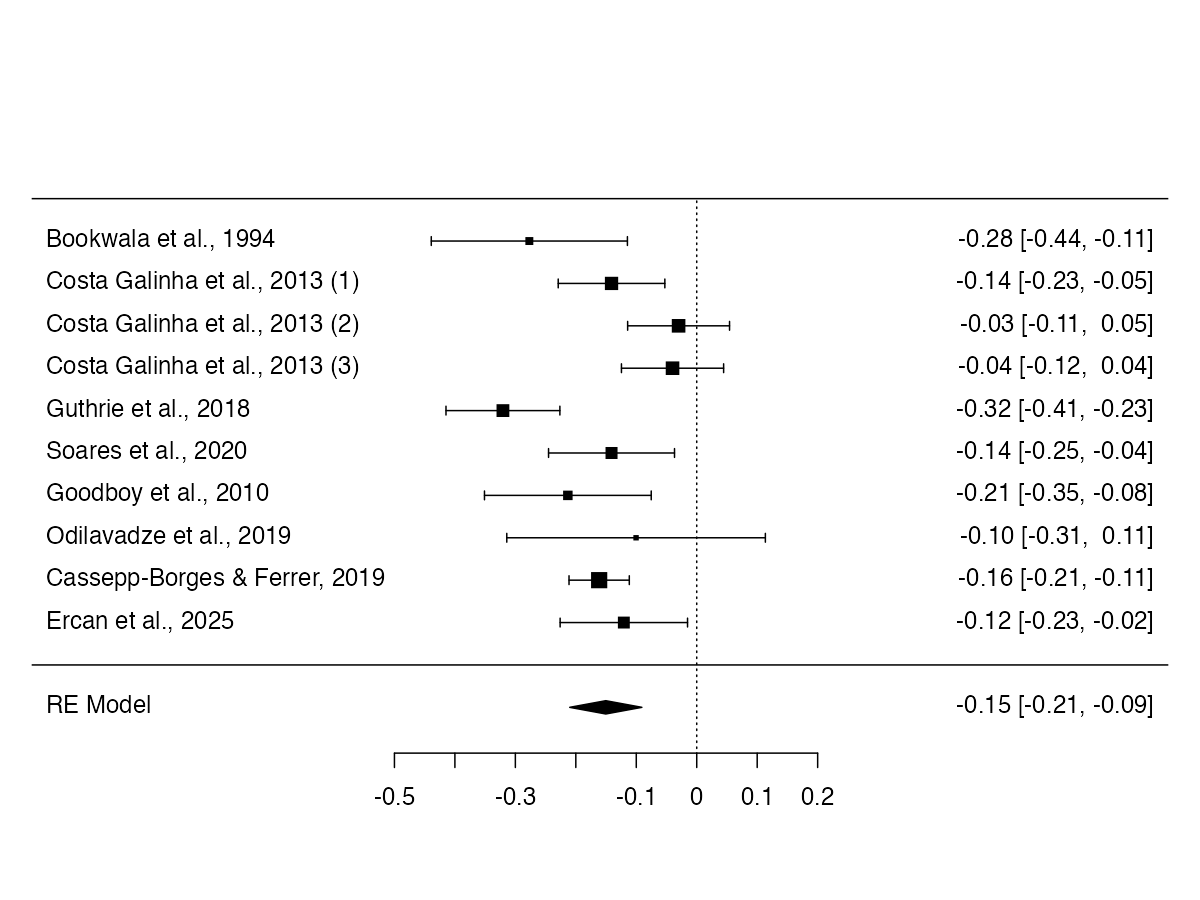


**Fig. 7S.**

*Forest plot for the association between emotional dependence and self-esteem.*


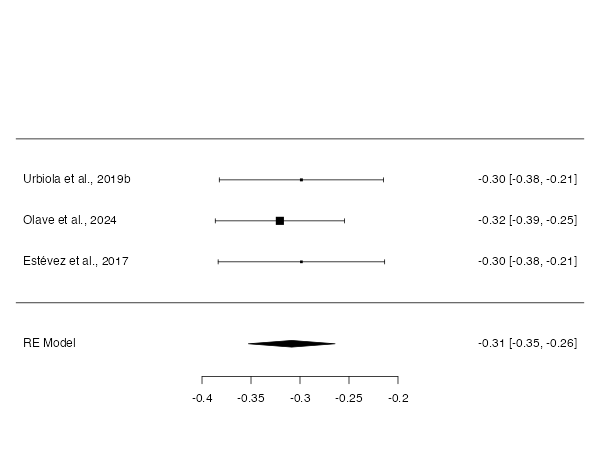


**Fig. 8S.**

*Forest plot for the association between emotional dependence and alcohol use/abuse.*


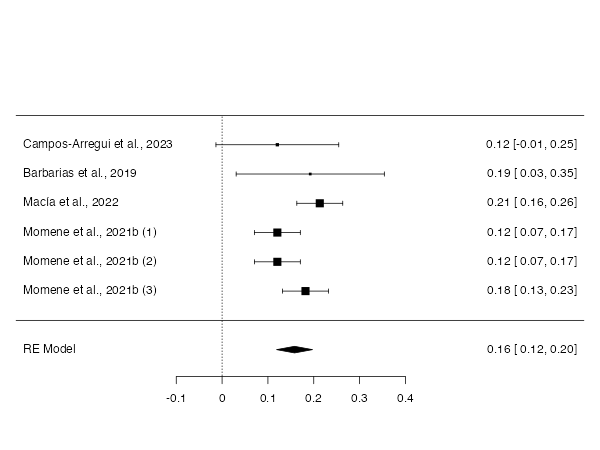


**Fig. 9S.**

*Forest plot for the association between emotional dependence and use/abuse of other substances.*


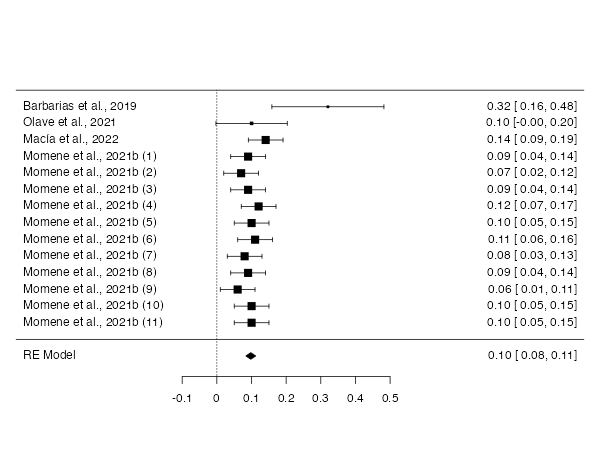


**Fig. 10S.**

*Forest plot for the association between emotional dependence and behavioral addiction.*

## *
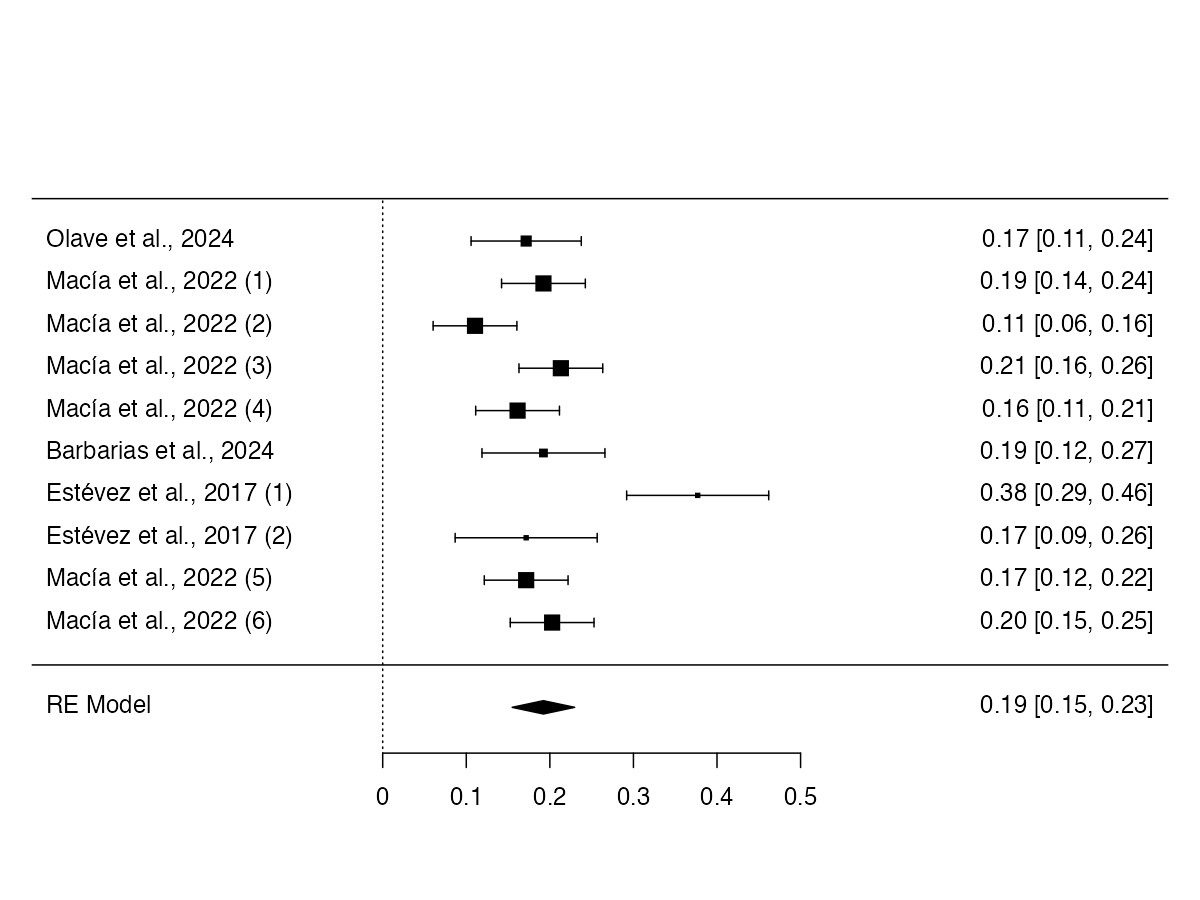
*

**Fig. 11S.**

*Forest plot for the association between emotional dependence and anxious attachment.*


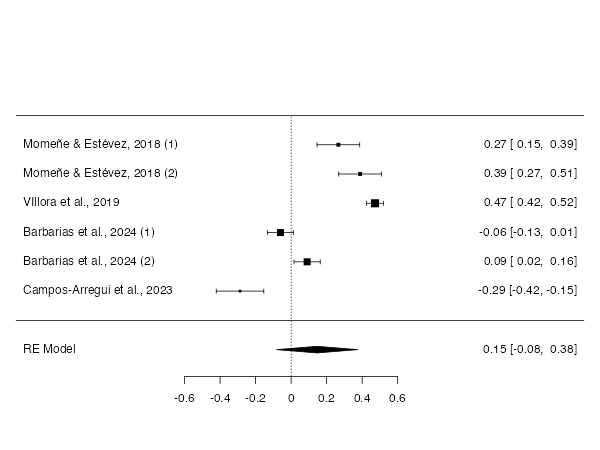


**Fig. 12S.**

*Forest plot for the association between emotional dependence and avoidant attachment.*


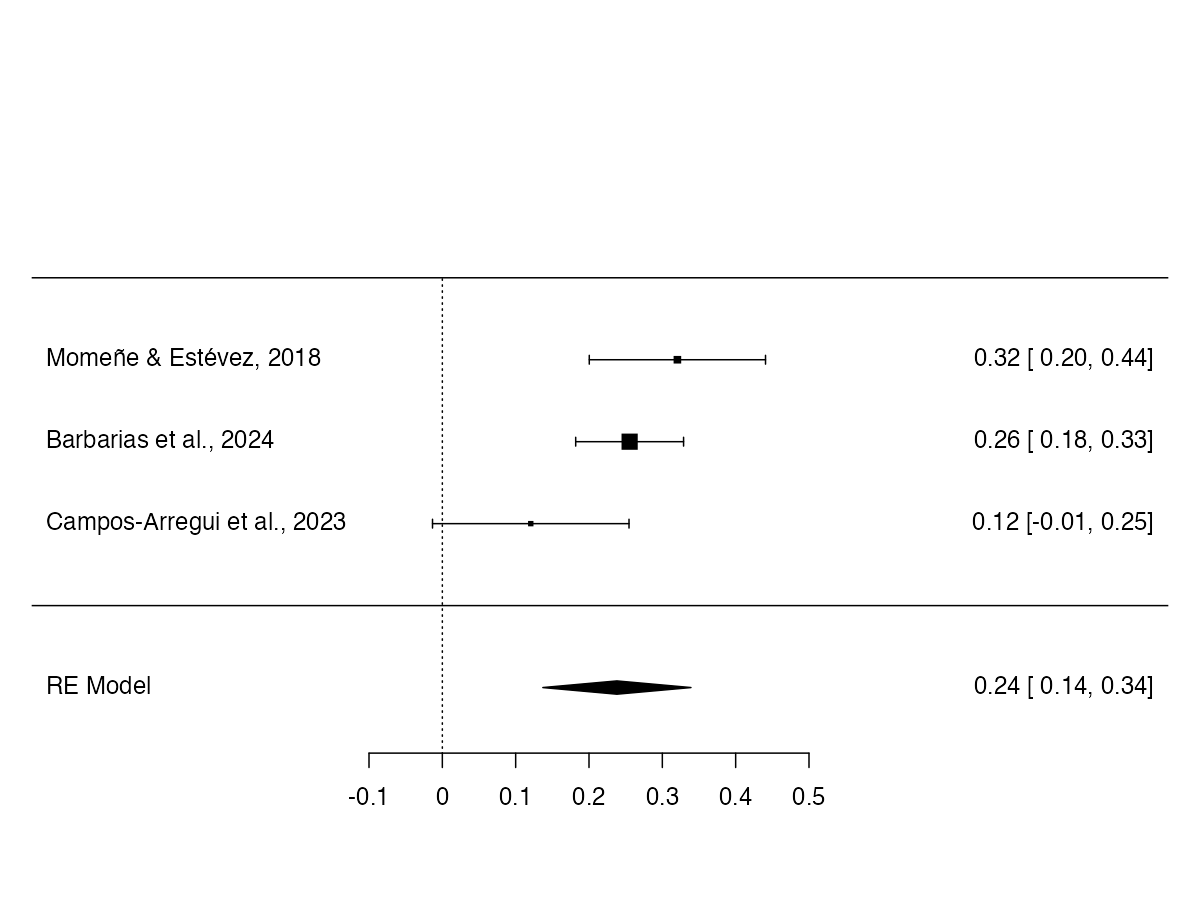


**Fig. 13S.**

*Forest plot for the association between emotional dependence and psychological violence received.*


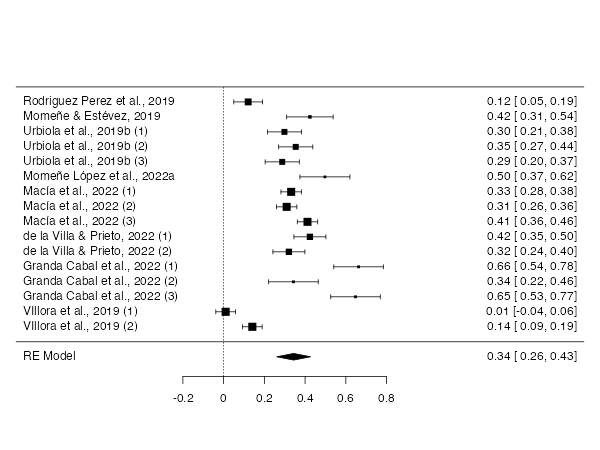


**Fig. 14S.**

*Forest plot for the association between emotional dependence and physical violence received.*


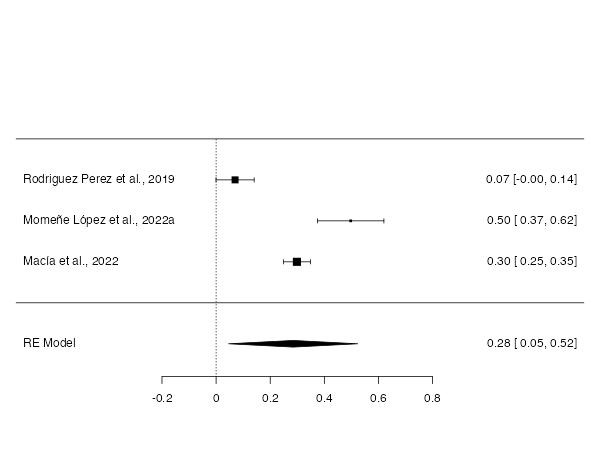


**Fig. 15S.**

*Forest plot for the association between emotional dependence and violence perpetrated.*


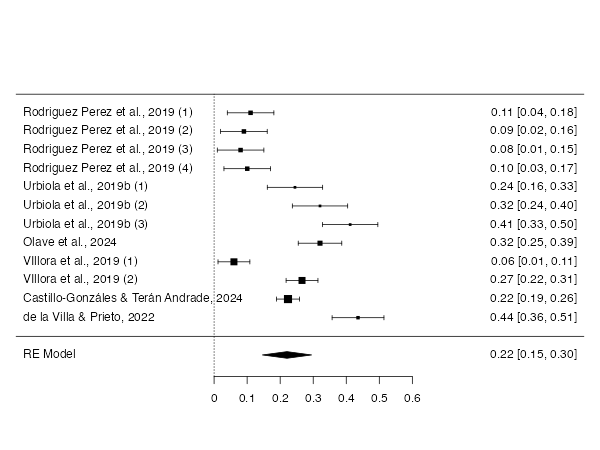


**Fig. 16S.**

*Forest plot for the association between emotional dependence and relationship satisfaction.*


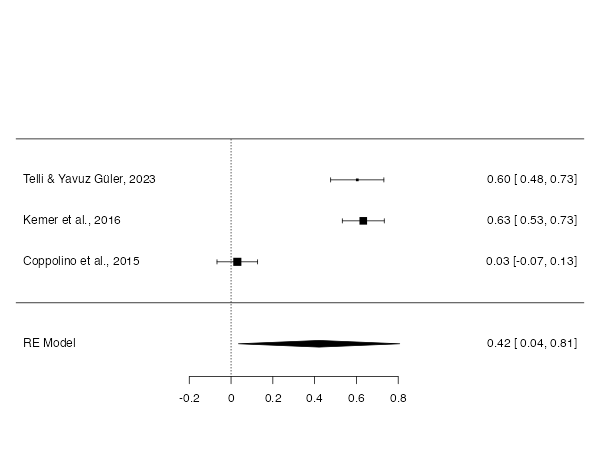


**Fig. 17S.**

*Forest plot for the association between love addiction and behavioral addiction.*


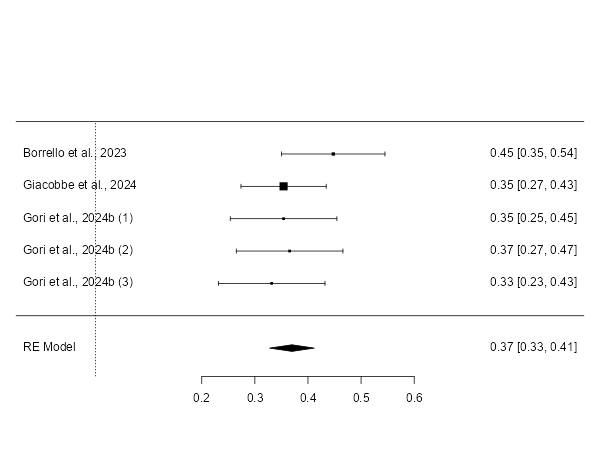


**Fig. 18S.**

*Forest plot for the association between love addiction and anxious attachment.*

## *
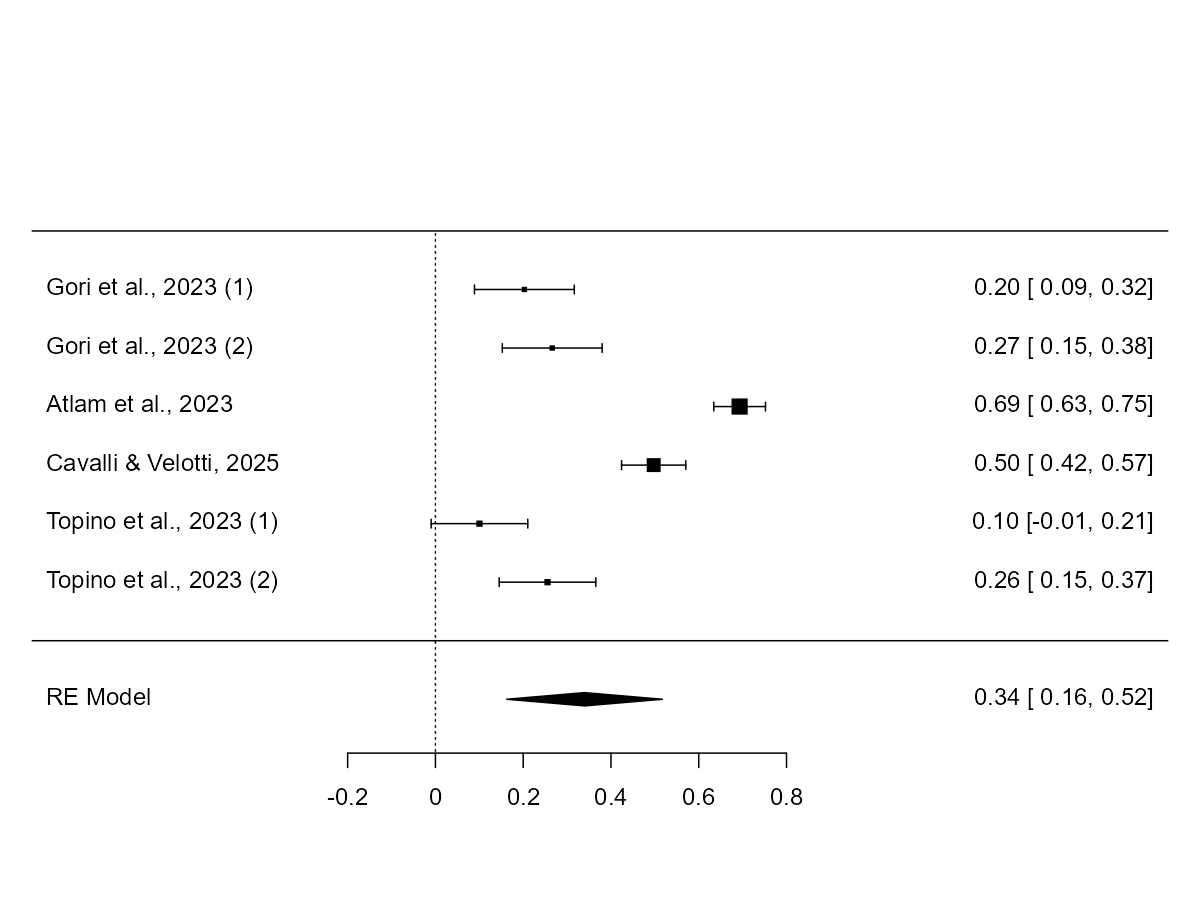
*

**Fig. 19S.**

*Funnel plot for the association between manic love and self-esteem.*


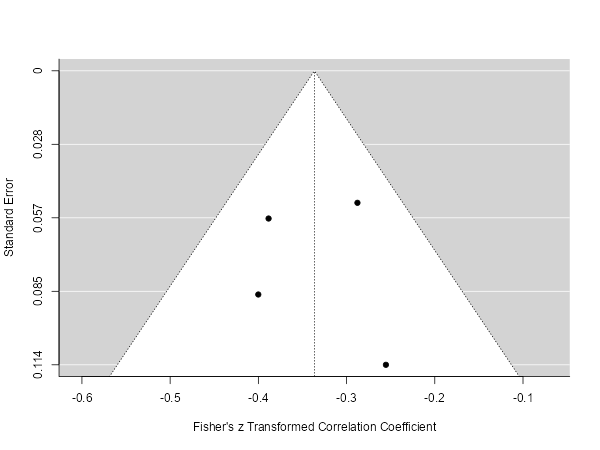


**Fig. 20S.**

*Funnel plot for the association between manic love and narcissism.*


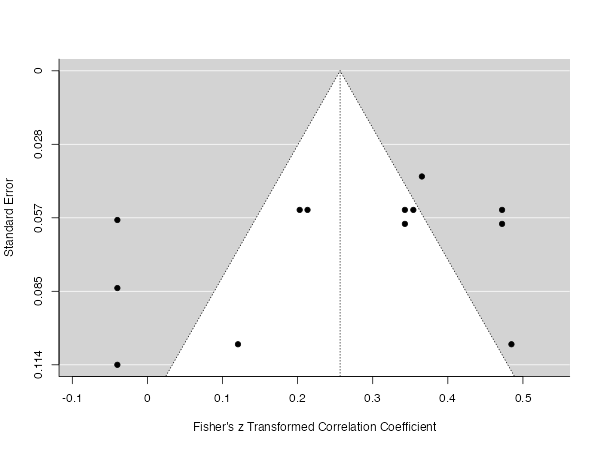


**Fig. 21S.**

*Funnel plot for the association between manic love and psychopaty.*


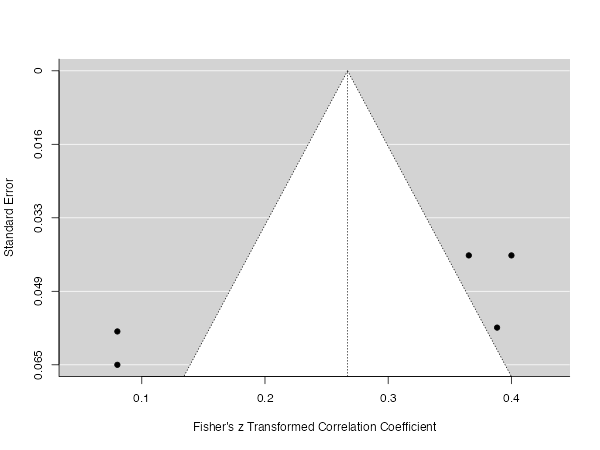


**Fig. 22S.**

*Funnel plot for the association between manic love and jealousy.*


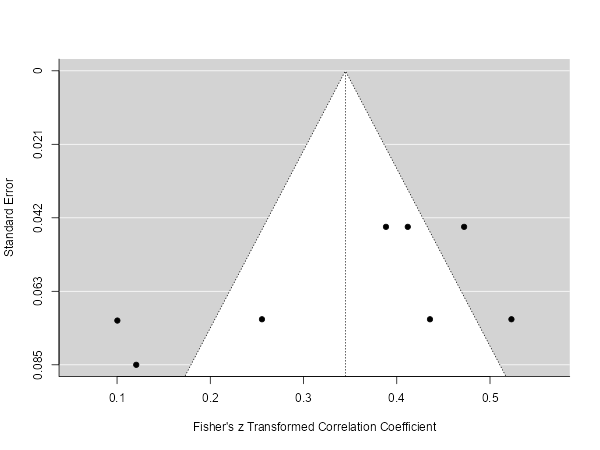


**Fig. 23S.**

*Funnel plot for the association between manic love and violence perpetrated.*


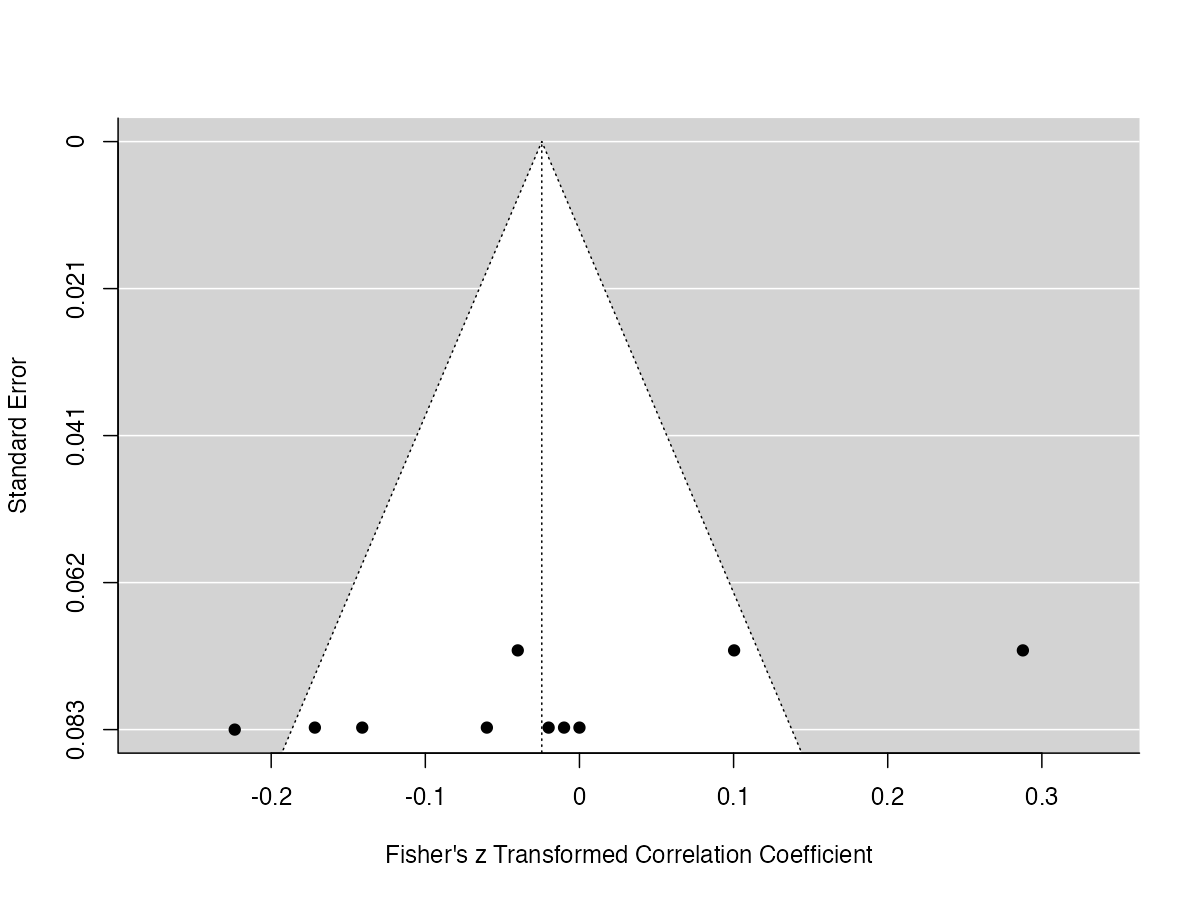


**Fig. 24S.**

*Funnel plot for the association between manic love and relationship satisfaction.*


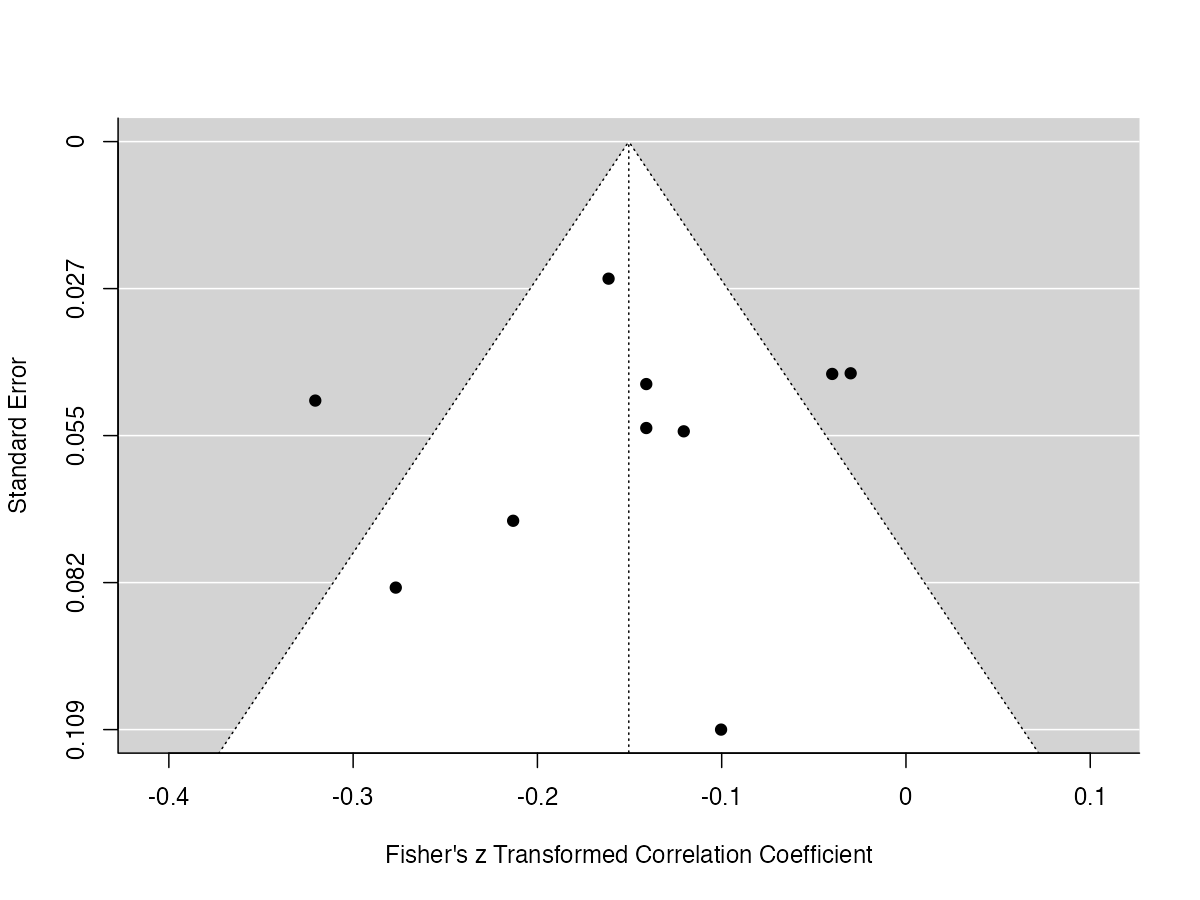


**Fig. 25S.**

*Funnel plot for the association between emotional dependence and self-esteem.*


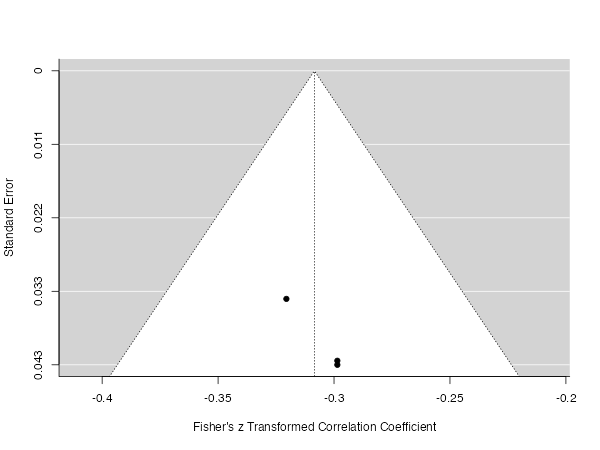


**Fig. 26S.**

*Funnel plot for the association between emotional dependence and alcohol use/abuse.*


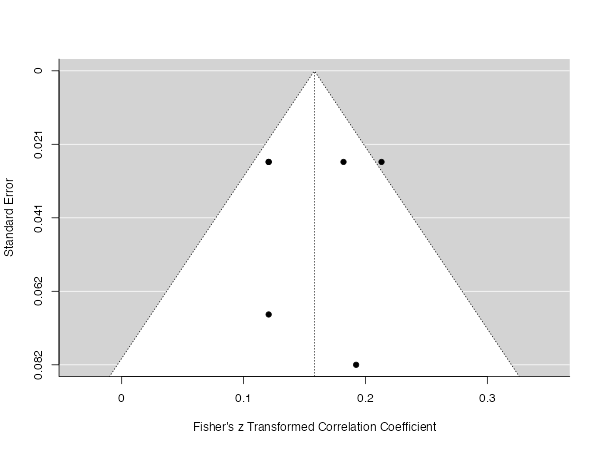


**Fig. 27S.**

*Funnel plot for the association between emotional dependence and use/abuse of other substances.*


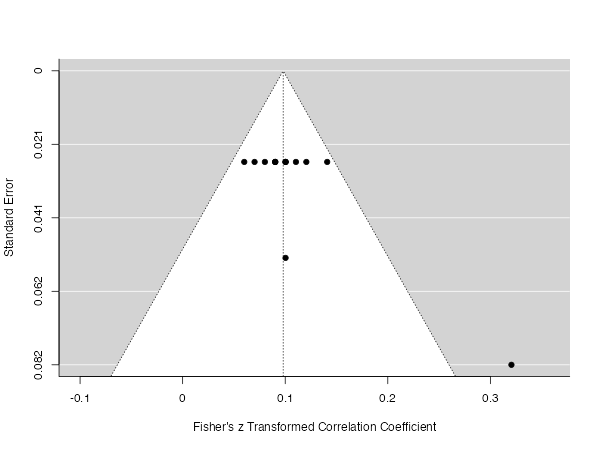


**Fig. 28S.**

*Funnel plot for the association between emotional dependence and behavioral addiction.*


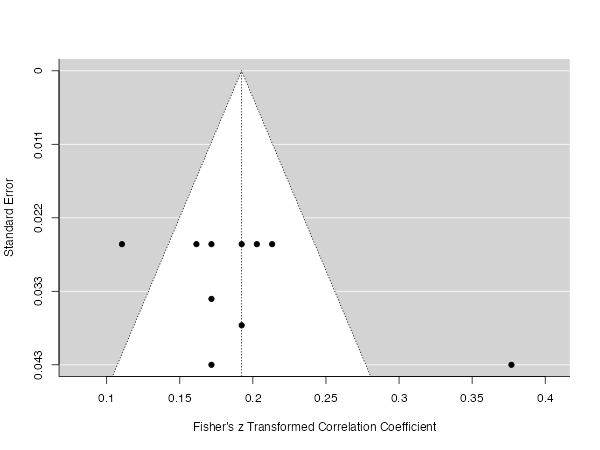


**Fig. 29S.**

*Funnel plot for the association between emotional dependence and anxious attachment.*


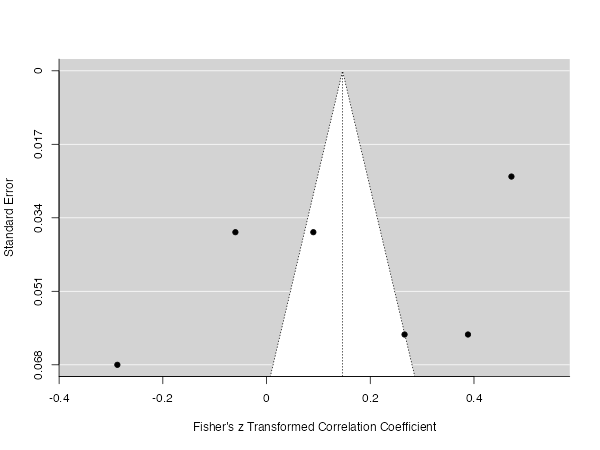


**Fig. 30S.**

*Funnel plot for the association between emotional dependence and avoidant attachment.*


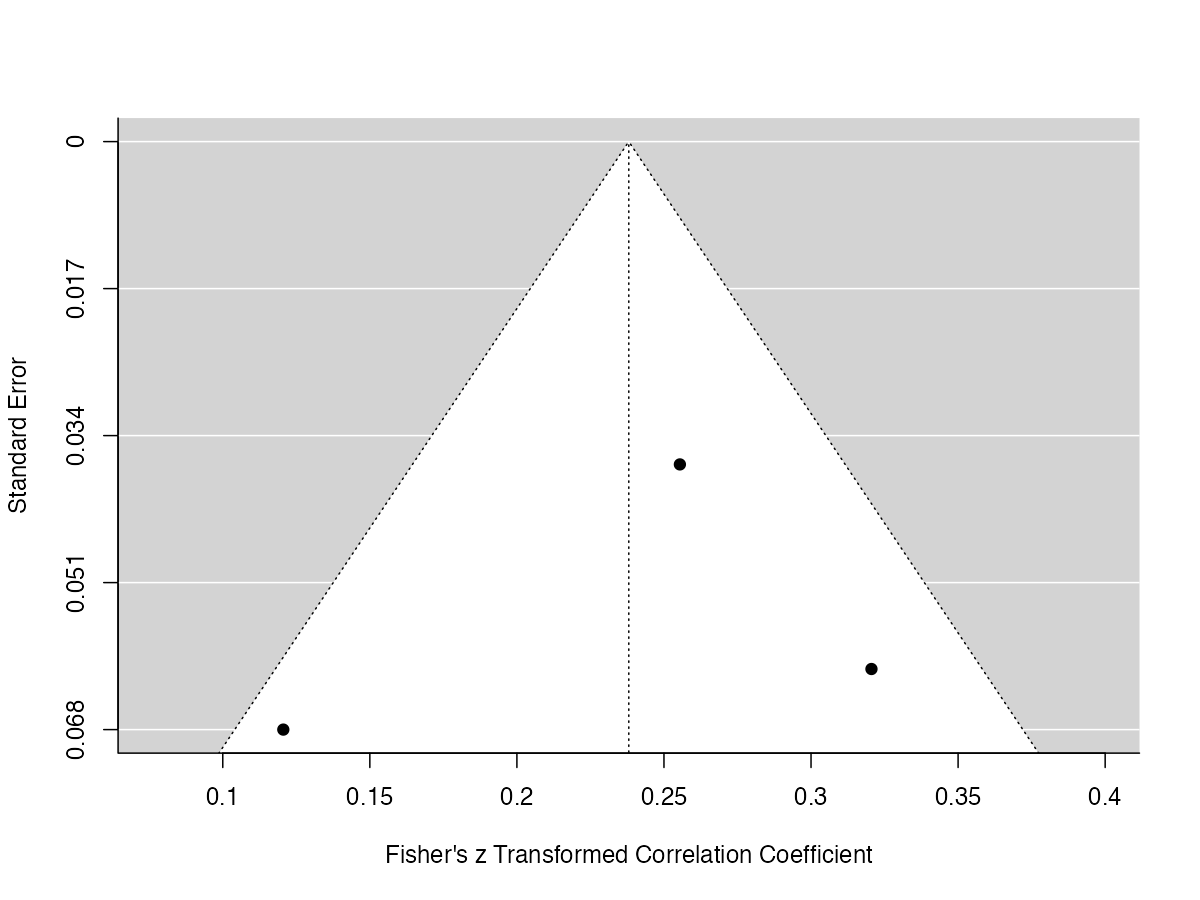


**Fig. 31S.**

*Funnel plot for the association between emotional dependence and psychological violence received.*


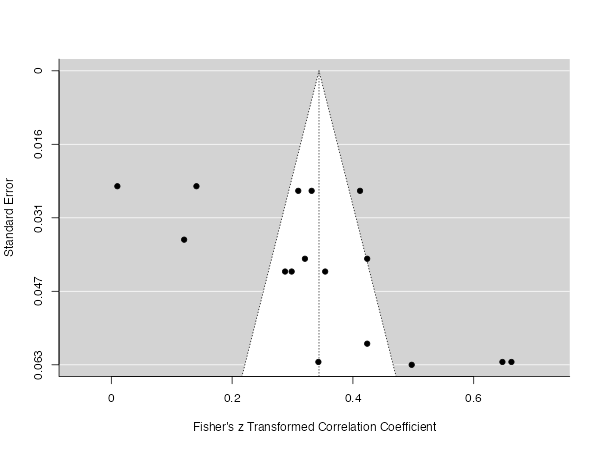


**Fig. 32S.**

*Funnel plot for the association between emotional dependence and physical violence received.*


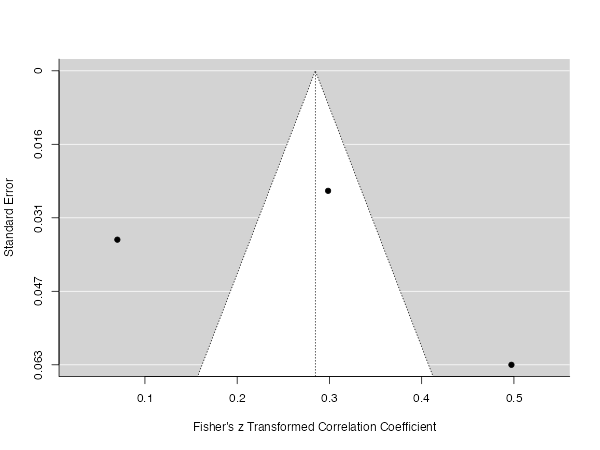


**Fig. 33S.**

*Funnel plot for the association between emotional dependence and violence perpetrated.*


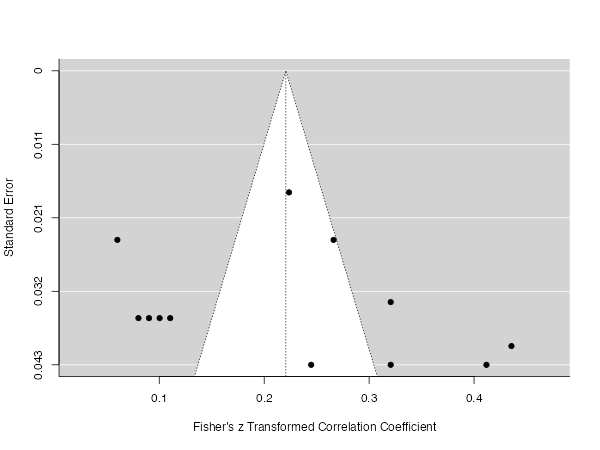


**Fig. 34S.**

*Funnel plot for the association between emotional dependence and relationship satisfaction.*


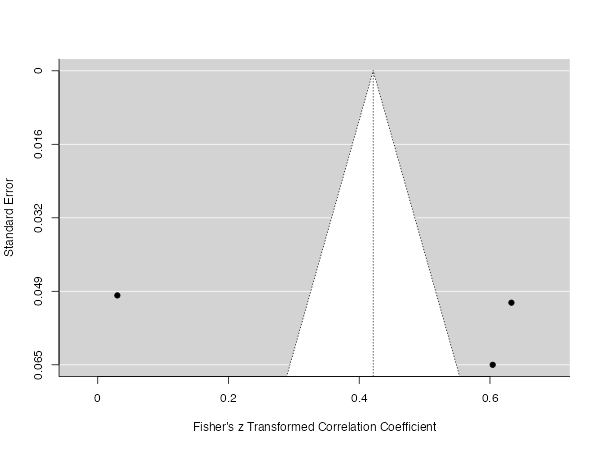


**Fig. 35S.**

*Funnel plot for the association between love addiction and behavioral addiction.*


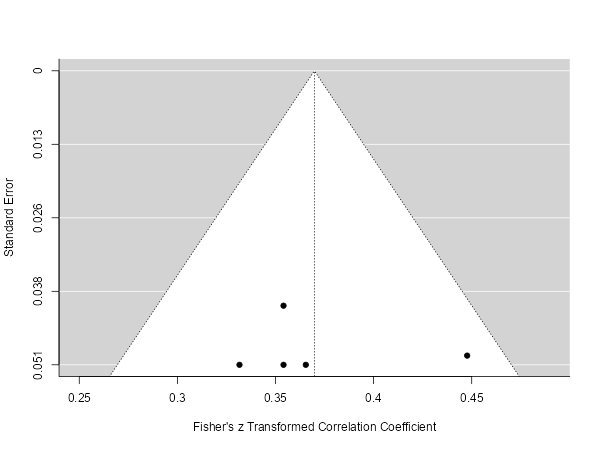


**Fig. 36S.**

*Funnel plot for the association between love addiction and anxious attachment.*

##
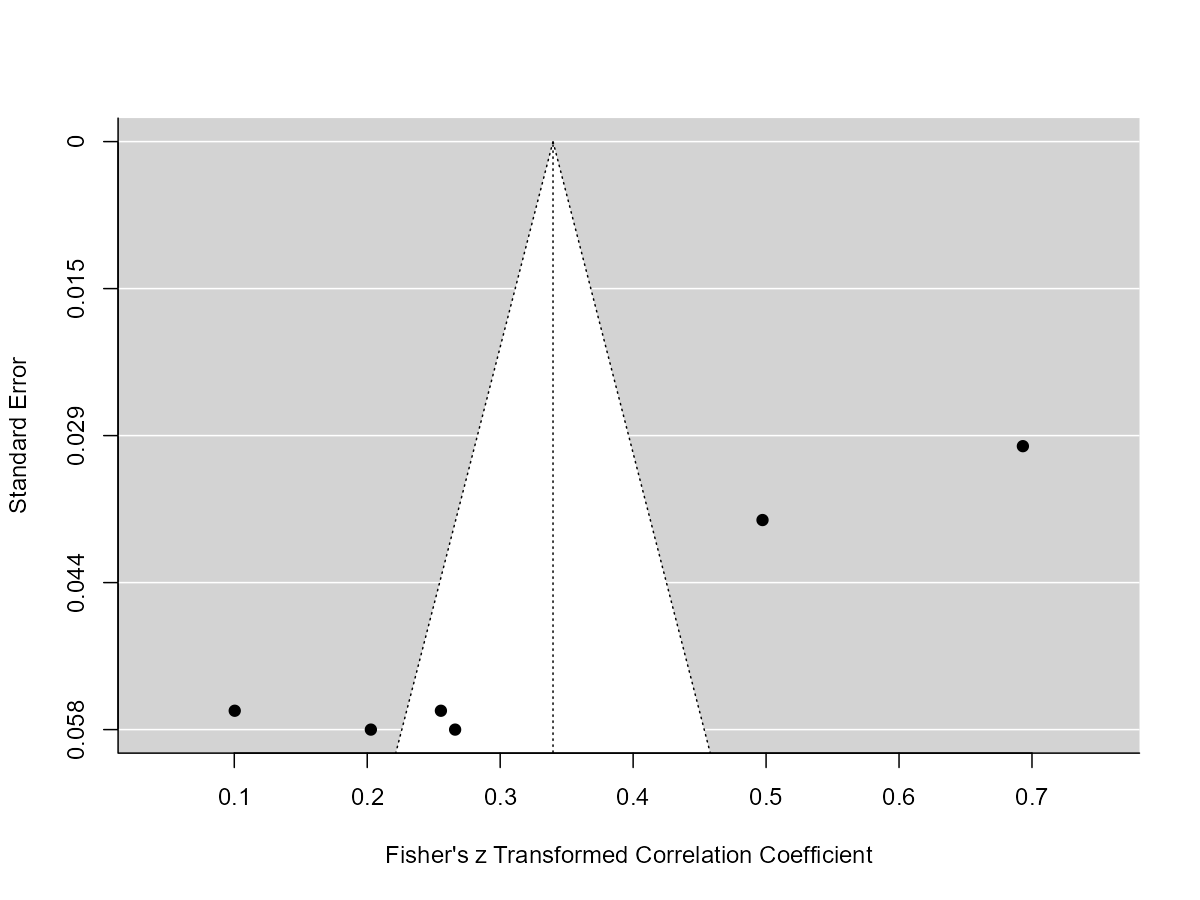

Supplement: Supplementary file 1 — Supplementary file1 (DOCX 6482 kb) [file 10508_2026_3420_MOESM1_ESM.docx]
